# Computational Mapping of Dirhodium(II) Catalysts

**DOI:** 10.1002/chem.202003801

**Published:** 2021-01-12

**Authors:** Adam I. Green, Christopher P. Tinworth, Stuart Warriner, Adam Nelson, Natalie Fey

**Affiliations:** ^1^ School of Chemistry and Astbury Centre for Structural Molecular Biology University of Leeds Leeds LS29JT UK; ^2^ GlaxoSmithKline Medicines Research Centre Stevenage SG12NY UK; ^3^ School of Chemistry University of Bristol Cantock's Close Bristol BS81TS UK

**Keywords:** computational chemistry, data-led prediction, homogeneous catalysis, ligands, rhodium

## Abstract

The chemistry of dirhodium(II) catalysts is highly diverse, and can enable the synthesis of many different molecular classes. A tool to aid in catalyst selection, independent of mechanism and reactivity, would therefore be highly desirable. Here, we describe the development of a database for dirhodium(II) catalysts that is based on the principal component analysis of DFT‐calculated parameters capturing their steric and electronic properties. This database maps the relevant catalyst space, and may facilitate exploration of the reactivity landscape for any process catalysed by dirhodium(II) complexes. We have shown that one of the principal components of these catalysts correlates with the outcome (e.g. yield, selectivity) of a transformation used in a molecular discovery project. Furthermore, we envisage that this approach will assist the selection of more effective catalyst screening sets, and, hence, the data‐led optimisation of a wide range of rhodium‐catalysed transformations.

## Graphical abstract

**A knowledge base** for dirhodium(II) catalysts, capturing their stereoelectronic properties, maps the relevant catalyst space and supports catalyst selection.

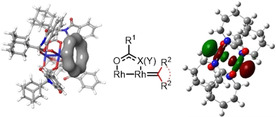

## Introduction

Some metal‐catalysed coupling reactions, such as the Suzuki and Buchwald–Hartwig reactions, are widely used to underpin the discovery of biologically active small molecules.^[1]^ However, many other powerful metal‐catalysed transformations are not part of the narrow toolkit of robust transformations that is widely exploited in drug discovery^[1]^ and development.^[2]^ The reluctance to utilise a wider range of metal‐catalysed reactions to drive discovery may stem from insufficient knowledge of substrate scope, and the perceived low likelihood of successful reaction outcomes with functionalised substrates.^[3]^

Approaches that enable the rapid optimisation of challenging catalytic reactions may promote the adoption of a broader reaction toolkit to drive drug discovery. Recently, the high‐throughput investigation of alternative catalyst systems for reactions involving hundreds of substrate pairs has been integrated into discovery workflows.[^4^, ^5^] In addition, design of experiment (DoE) approaches, in which ligand property descriptors guide ligand selection,^[6]^ have been exploited in the optimisation of a diverse range of challenging reactions including specific Suzuki, Heck, Buchwald–Hartwig, Ullmann and borrowing hydrogen‐mediated *N*‐alkylation reactions.^[7]^

The chemistry of rhodium carbenes is extremely diverse, and includes insertion into C−H, N−H and O−H bonds, cyclopropanation and ylid formation.^[8]^ As a result, Rh catalysis can enable the exploration of diverse chemical space and, hence, the discovery of many different classes of bioactive small molecule.^[9]^ The reactions catalysed by dirhodium(II) complexes can often be tuned through ligand effects, allowing high levels of chemo‐, regio‐ and stereoselectivity to be imparted in many different contexts.^[10]^ As a result, careful ligand selection can provide exquisite control over the outcome of reactions that have many possible outcomes.^[10]^ This diversity, however, makes detailed mechanistic studies challenging, for example because catalyst modification can affect the selectivity between alternative reaction pathways including transition state bifurcations.^[11]^ In such cases, accurate computational predictions are difficult,^[12]^ meaning that, in general, only a few catalysts can be evaluated mechanistically ahead of an experimental programme.^[13]^ Capturing the impact of catalyst diversity on complex reaction pathways would thus benefit from a catalyst selection approach that was independent of reaction mechanism, with parameters chosen to be representative and transferable across the entire spectrum of reactivity.

In the domain of dirhodium(II) catalysts used for C−H functionalisation, approaches to catalyst parameterisation have used calculated steric and electronic parameters for the substrate^[10f]^ or a surrogate,^[10e]^ as well as structural analysis of the interplay between catalyst and substrate structure.^[10e]^ In such cases, careful selection of parameters could enable high correlation with experimentally‐determined free energy differences, supporting the derivation of predictive models specific to individual reactions. In addition, substrate chemical space has been explored further for reactions of α‐diazo β‐carbonyl esters,^[10b]^ where individual steric and electronic parameters for substrates have been related to both calculated enthalpies of activation for insertion into C(sp^3^)−H bonds and a range of experimentally‐measured yields and selectivities. By necessity, the models arising from these studies are reaction‐specific and focussed on the substrate; we further note that selectivity prediction poses significant computational challenges.[^12b^, ^14^]

Predictive models built using reaction‐specific parameters, that is, those describing catalytic intermediates,^[15]^ could bias models towards known reaction pathways, making them ineffective when a catalyst modification triggers a deviation from the captured reactivity.^[16]^ We were therefore interested in mapping dirhodium(II) catalyst properties, and relating these to observed catalyst reactivity. We have previously developed ligand databases for both monodentate *P*‐donor^[17]^ and chelating *P,P*‐ and *P,N*‐donor^[18]^ ligands by principal component analysis (PCA) of steric and electronic parameters calculated with a standard DFT approach (for a broader review of this area, see reference [6]). These so‐called knowledge bases can facilitate the optimisation of diverse challenging metal‐catalysed reactions without recourse to mechanistic data.^[17b]^ Here, we describe the development of a database for dirhodium(II) catalysts which shares this pragmatic philosophy. We show that the database could have facilitated the design of reaction arrays that underpinned a bioactive molecular discovery project. In the longer term, we envisage that it may also facilitate the optimisation of diverse Rh‐catalysed reactions in discovery and other contexts, supporting the design of diverse catalyst screening sets, the visualisation of outcomes and how they relate to catalyst properties, as well as further optimisation if a cluster of active catalysts can be detected.

## Results and Discussion

### Design of a database of descriptors for dirhodium(II) catalysts

**Scheme 1:**
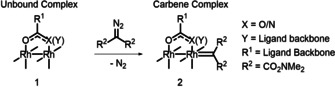
Dirhodium complexes modelled using standard DFT (BP86/6‐31G(d), with MWB28 on Rh).

**Figure 1:**
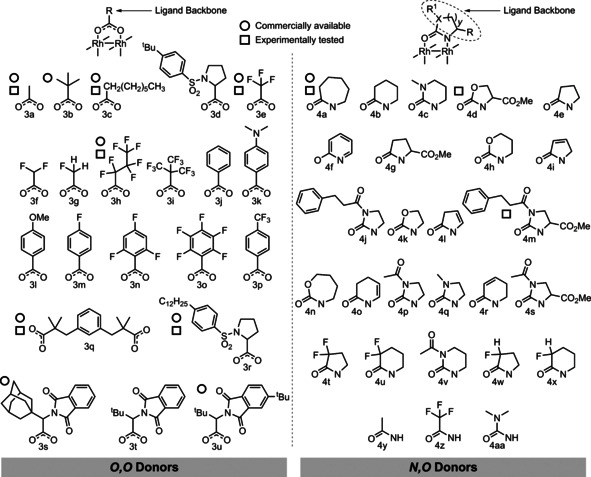
The 48 ligands in the dirhodium(II) complexes that were used to generate the database. Ligands are drawn with coordinating atoms at the bottom. The labels correspond to the corresponding dirhodium(II) complexes bearing these ligands. Catalysts that are commercially available (circle) or that were investigated in high‐throughput experiments (square) are indicated.

We sought to capture chemically relevant information about dirhodium(II) complexes that would be independent of the mechanisms of their specific reactions. Structures were calculated, using DFT (see Supporting Information for full computational details), for the unbound dirhodium(II) complexes **1** and the representative carbene complexes **2** generated from a symmetrical α‐diazo malonamide precursor (Scheme 1). This acceptor–acceptor carbene acts as a reporter for the effect of changes in the catalyst structure on a model substrate, and we note that other types of carbene could have fulfilled the same function. We calculated structures for the dirhodium(II) complexes **1** and **2** bearing 48 different bidentate ligands (Figure 1) using the standard BP86 functional^[19]^ with the 6‐31G(d) basis set^[20]^ on all atoms apart from rhodium, where the MWB28 effective‐core potential basis set was used^[21]^ (see Supporting Information for full computational details and comments on the exploration of conformational space). Here, structural and electronic parameters (note that we use the term descriptors interchangeably) were obtained for dirhodium(II) complexes with a wide range of bidentate carboxylate (*O*,*O*) or carboxamidate (*N*,*O*) ligands (Table 1).

**Table 1:** Calculated descriptors for the catalyst database, data included in Supporting Information.

Descriptor	Derivation	Diagram
*r*(Rh‐Rh)	Rh−Rh bond length (Å), **1** and **2**	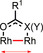
∡(Rh‐Rh‐L)	average Rh−Rh−ligand bond angle, **1** and **2**	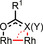
∡(O‐C‐X)	average ligand bite angle, **1**	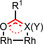
∡(C−C‐C)	carbene C−C−C angle, **2**	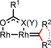
HOMO, **1**	energy of HOMO for complex **1** (a.u.)	Supporting Information Figures S3–S5
LUMO, **1**	energy of LUMO for complex **1** (a.u.)	Supporting Information Figures S3–S5
Q Rh, **1**	charge on rhodium atoms	–
Q(L, mean), **1**	Mean charge on ligands	–
Δ*E*(FMO)	Δ*E* between HOMO and LUMO (a.u.)	–
		
|wV|	distance‐weighted volume,^[22]^ **1**	$V_{W,\;k,\;l\;}=\;\sum_{i-1}^{n}\frac{r_{i}^{k}}{d_{i}^{l}}$
		
He_8_	interaction energy for **1** and ring of 8 helium atoms^[17]^ [kcal mol^−1^]	Figure 2, He_8_=E(He_8_⋅[Rh‐Rh]))−E(He_8_)−E([Rh‐Rh]))
Δ*E*(coord)	energy for diazo precursor to form the carbene complex [kcal mol^−1^], **2** (Scheme 1)	Δ*E*(coord)=(E**1**+E**Diazo**)−(E**2**+E**N_2_**)

The parameters were chosen to capture the range of possible electronic and steric effects arising from different ligand types in this coordination environment. Parameter selection for inclusion in the final principal component analysis is explored more fully in the Supporting Information. The ligands were selected or designed to ensure an even distribution of features such as donor atom types and substitution patterns, and synthetic feasibility was also considered (for additional parameters that were captured, but ultimately not used, see Supporting Information).

Ligands with modifications such as fluorination (**3 e**–**3 i**, **4 t**,**u** and **4 w**–**4 x**), unsaturation (**4 i**, **4 l**, **4 o** and **4 r**) and heteroatom substitution (e.g. **4 d**, **4 h**, **4 k**, **4 n** and **4 q**) were designed to investigate the electronic effect of the ligand backbone on the complex. Ligands with different cyclic carboxamidates were also included to investigate structural changes induced by ring size (**4 a**–**4 x**). Several ligands with substituents which may intrude into the metal coordination sphere (e.g. **3 r**–**3 u** and **4 d**, **4 g**, **4 m** and **4 s**) were included because such catalysts have been reported to enable highly selective insertions into some unactivated C−H bonds.^[10d]^

**Figure 2:**
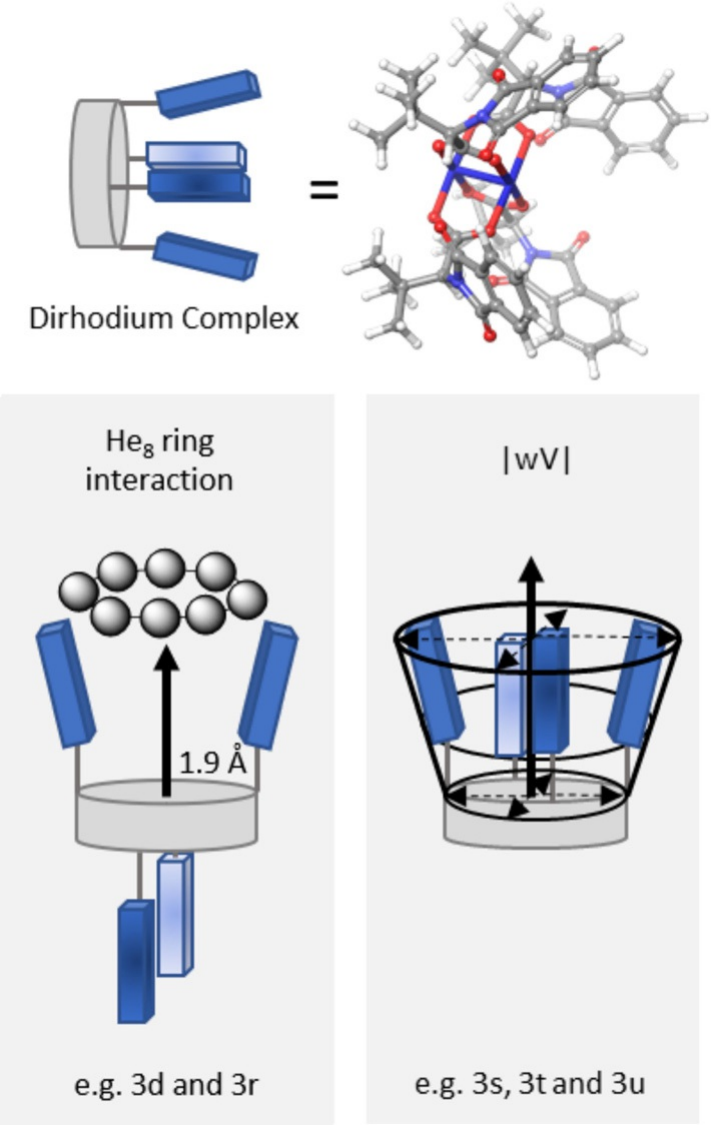
Steric parameters captured. The He_8_ ring was positioned 1.9 Å from the Rh atom forming the carbene bond.^[17b]^ |wV| was aligned along the vector of the Rh‐Rh bond^[22]^ (see Figure S2† for examples).

A wide‐range of descriptors was calculated to capture the electronic and steric properties of each dirhodium(II) complex, and the set of 14 descriptors (listed in Table 1) were chosen based upon the quality of clustering observed in the top‐performing PCA models (for data and full details of descriptor selection, see Supporting Information). Features that each descriptor captures can be classified into three categories: descriptors capturing electronic properties (HOMO, **1**, LUMO, **1**, Q Rh, Q(L, mean) and Δ*E*(FMO)); descriptors capturing a mixture of electronic and steric properties (*r*(Rh‐Rh), ∡(Rh‐Rh‐L), ∡(O‐C‐X), ∡(C−C‐C) and Δ*E*(coord)); and descriptors capturing steric properties (He_8_ and |wV|). |wV|^[22]^ gives a measure of the steric bulk of a ligand by considering the proximity of the ligand to an important atom, in this case the rhodium atom bound to the carbene (Figure 2). The He_8_ descriptor also captures the steric influence of ligand substituents by modelling the approach of a reactant to a ring of eight helium atoms (Figure 2), a modified version of steric descriptors described previously.^[17]^ |wV| and He_8_ were calculated using the optimised geometry of the carbene complex, with the carbene ligand then removed and He_8_ aligned with the Rh−Rh bond.

**Figure 3:**
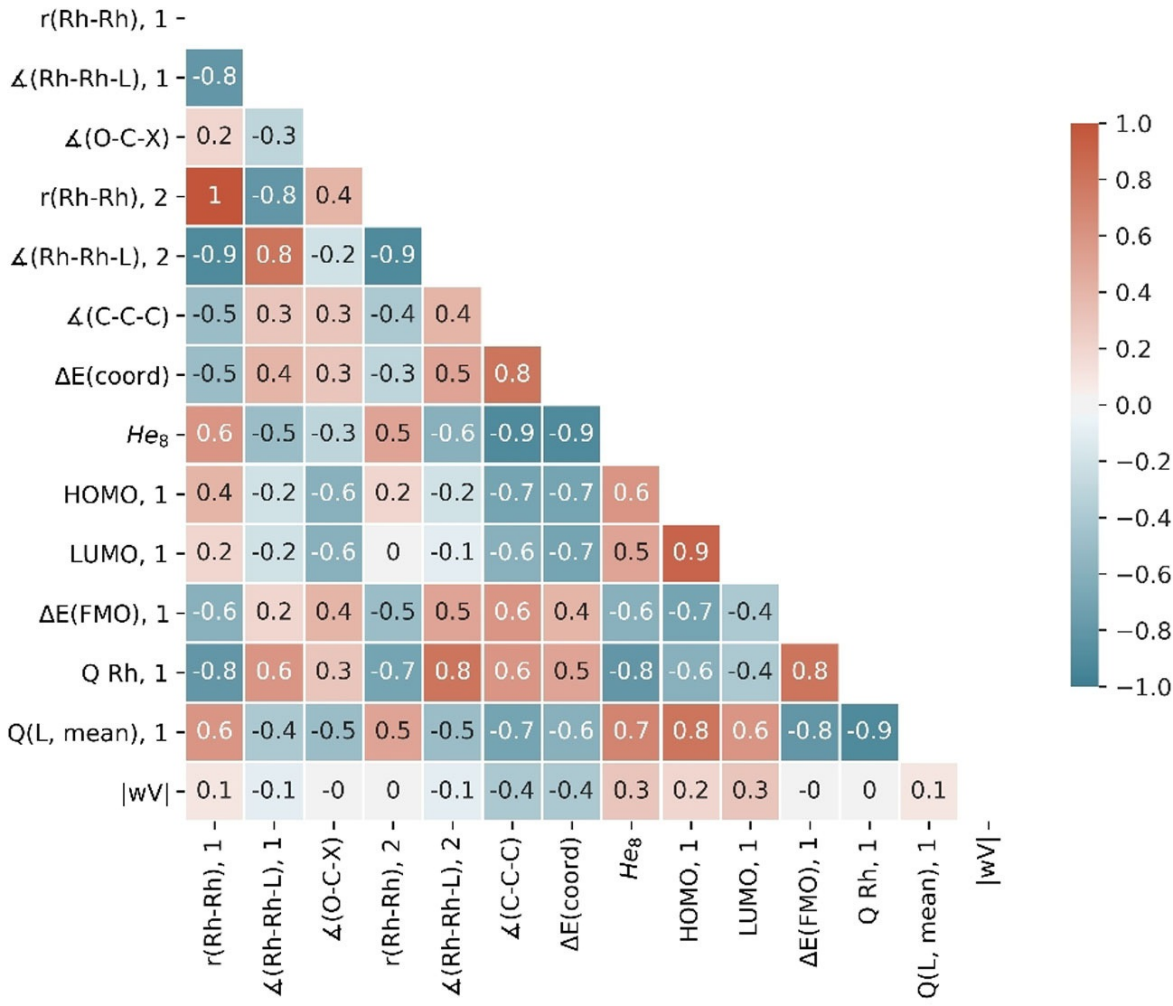
Calculated Pearson R correlation coefficient map for the 14 selected descriptors.

Correlation analysis was then performed on the dataset to evaluate the performance of our chosen parameters and to contextualise the information captured (Figure 3). Strong correlations between *r*(Rh‐Rh) and ∡(Rh‐Rh‐L), with |R| values ranging from 0.8–1.0, reflect the rigidity of the Rh_2_L_4_ scaffold. Changes to the Rh‐donor distances, either through electronic effects or steric clashes, affect the Rh‐Rh bonding, along with the geometry of the ligand coordination. A different form of steric effect, best described as the extent to which the ligand's substituents intrude in the site of reaction, is important for controlling the geometry of the metal carbene and the subsequent angle of attack of reactants; this likely gives control over regio‐ and stereochemistry.^[10]^ Catalysts **3 r**–**3 u** and **4 d**, **4 g**, **4 m** and **4 s** have the largest distance‐weighted volume, |wV|,^[22]^ values due to ligand projection over the axial face. Weak correlations between |wV| and all other parameters confirm that this is a unique and purely steric term, with |R| values ranging between 0 and 0.4. Electronic effects are also important, modulating the Lewis acidity of the complex and control the chemoselectivity of a reaction where two competing pathways occur.^[10j]^

**Figure 4:**
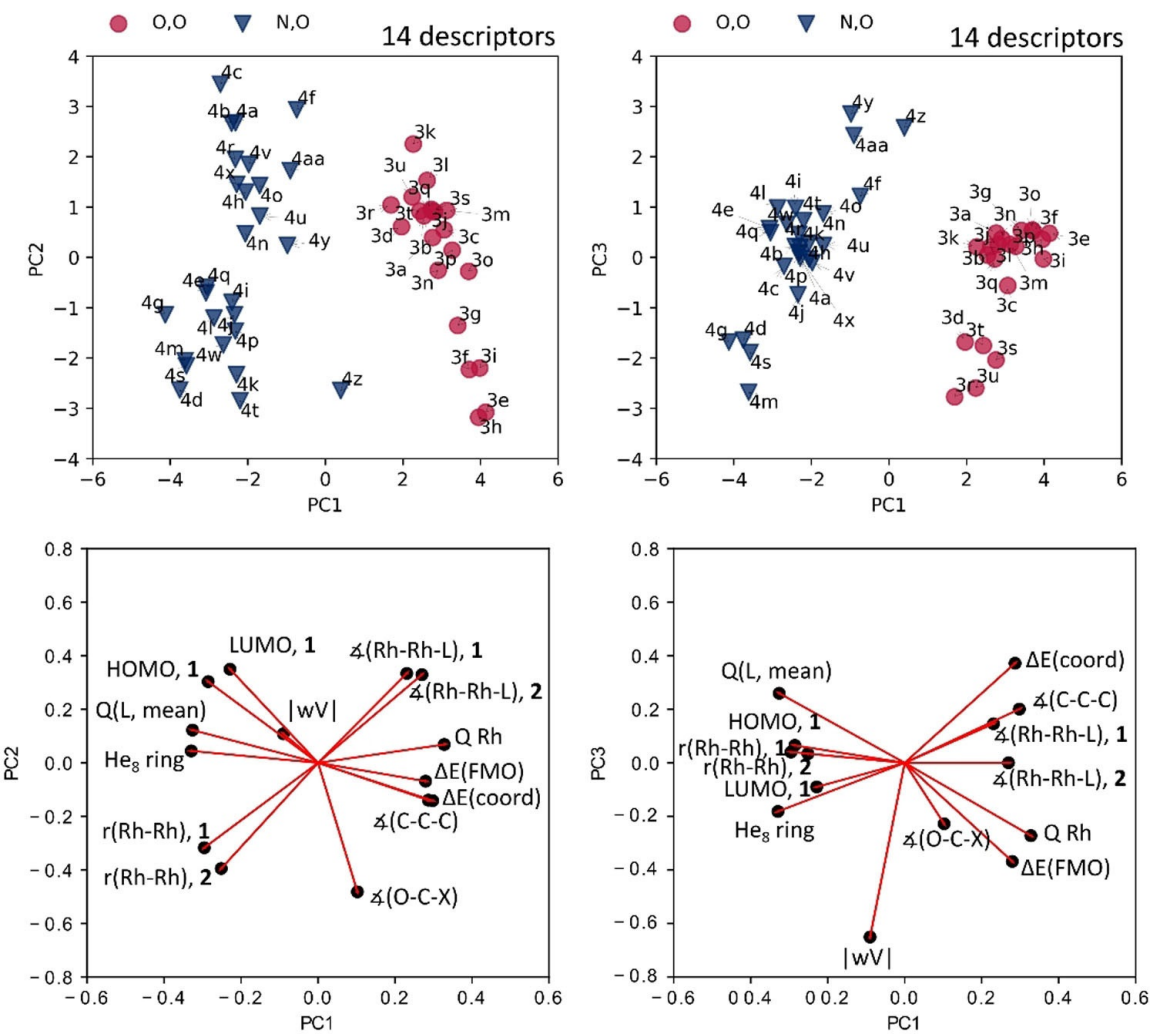
PCA score and loadings plots. Optimal solution for the PCA of dirhodium(II) catalysts capturing around 86 % total variance. PC1/PC2/PC3 explained variance: 54, 22 and 11 %. Mean squared error loss from projection: 0.142.

We performed a principal component analysis (PCA) using the 14 computationally‐derived parameters (Table 1, see Supporting Information for further details on parameter selection). PCA enables correlated variables to be converted into fewer principal components, that is, linear combinations of the original parameters that are orthogonal and capture most of the variation in the data.^[23]^ The first two principal components (PC1/PC2) capture 75 % of the combined variance in the dataset for all 48 catalysts, whilst the first three principal components capture 86 % of the combined variance (Figure 4, further details shown in Supporting Information, Figures S6–S12 and Tables S5–S7).

PCA is designed to pick up the largest sources of variation in the data, and PC1 alone separates most of the dirhodium complexes bearing carboxylate (**3**) and carboxamidate (**4**) ligands into distinct regions of the map, as expected from this approach. Increased fluorination of the carboxylate ligands leads to decreased PC2 values, suggesting that PC2 captures the Lewis acidity of the complexes. Furthermore, the ring size of carboxamidate ligands is also captured by PC2, producing clusters of complexes with 5‐ and 6/7‐membered carboxamidate ligands. The first three principal components capture around 86 % of the combined variance and separate the complexes with carboxylate and carboxamidate without intermixing; in addition, the five complexes with “intrusive” carboxylate ligands (**3 d**, **3 r**–**3 u**) and the four complexes with “intrusive“ carboxamidate ligands (**4 d**, **4 g**, **4 m** and **4 s**) form distinct clusters, along with a separate cluster for the carboxamidate ligands (**4 y**–**4 aa**) with acyclic backbones (Figure 4; see Supporting Information for descriptor loadings (Table S6) and eigenvalues (Table S7). We note that ligand **3 q**, which is the only ligand with two donor pairs, does not stand out in the PCA, suggesting that the tethered backbone does not contribute substantially to variation in this dataset.

Figure 4 also shows the loadings of individual descriptors in this PCA model and here we note that most descriptors load highly on all three principal components, with the exception of |wV|, which is only picked up on PC3. The correlations identified between descriptors (Figure 3) are also reflected in their loadings. Since PCA is not statistically robust, we will avoid a detailed interpretation to attach meaning to individual PCs, relying instead on the chemical interpretations of catalyst clustering set out above. Note, however, that PC1 loads parameters focussed on the metal centres highly, while PC2 captures ligand effects, with a particular focus on Lewis acidity. The distance‐weighted volume |wV|, which captures intrusion of ligands into the coordination sphere, along with electronic ligand‐based parameters, load quite highly on PC3, potentially helping to resolve data within the two main subsets.

### Relation of the catalyst map to a reaction exploited in bioactive molecule discovery

**Figure 5:**
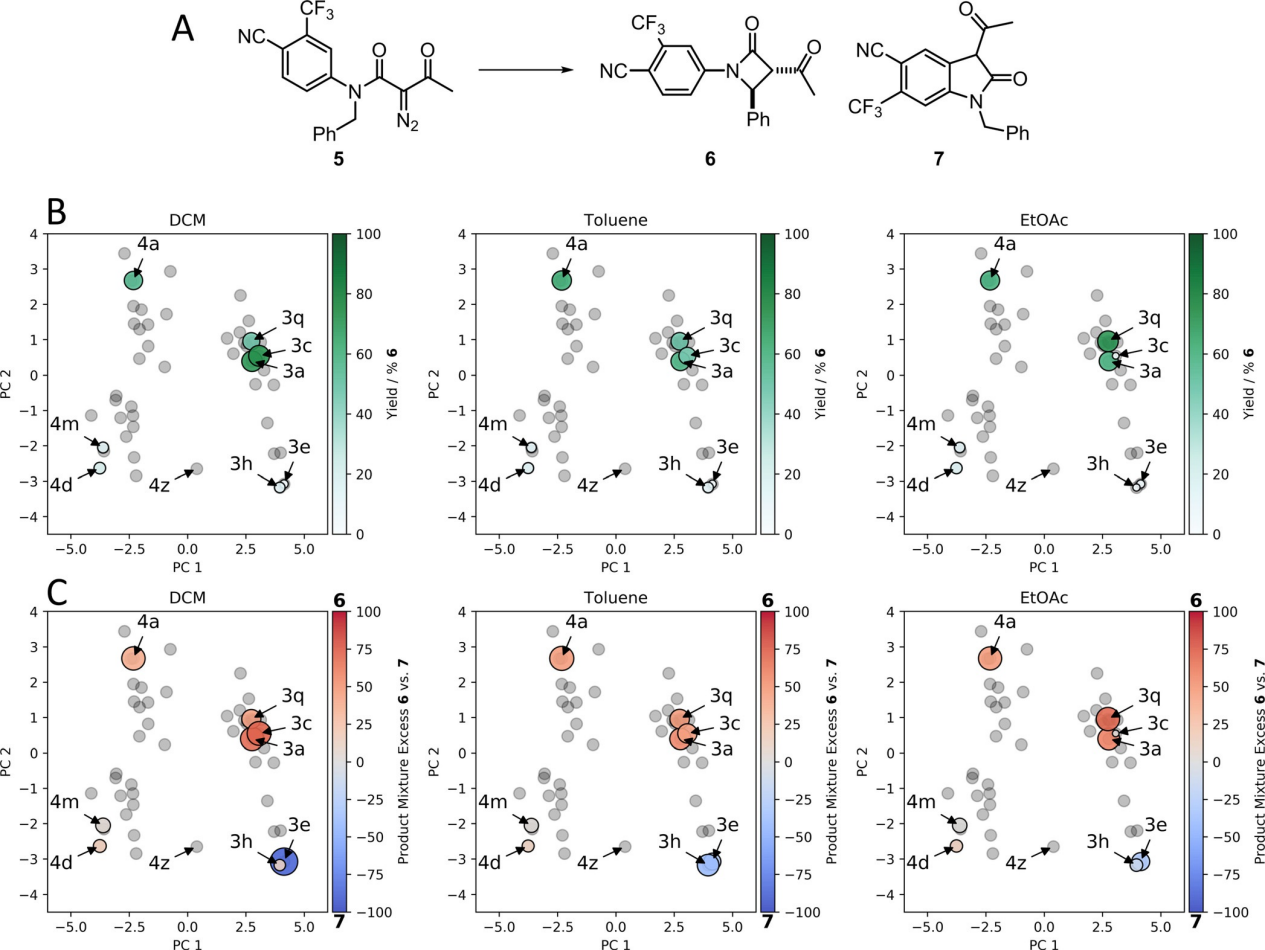
Effect of catalyst on the outcome of a reaction that underpinned the discovery of the androgen receptor agonist **6. Panel A**: Reaction overview. **Panel B**: HPLC yield of the β‐lactam **6** as a function of catalyst and solvent shown within the context of the catalyst map (green, circles scaled according to yield, with darker shades also corresponding to higher % yield of 6); catalysts that were not investigated experimentally are also shown (grey, **4 z** labelled). **Panel C**: Ratio of HPLC peak areas corresponding to the alternative products **6** and **7** (circles scaled according to conversion, with red favouring **6** and blue favouring **7**) (see Figure 1 for details of catalysts and Figures S13–S17 for PC1/PC3 maps and correlations).

Recently, the high‐throughput investigation of alternative catalyst systems has been integrated into drug discovery workflows.[^4^, ^5^] This practice has been prompted because, often, no individual catalyst system is best for all pairs of functionalised substrates. In a project directed towards the discovery of androgen receptor modulators,^[9a]^ we harnessed Rh‐catalysed reactions of diazo amides because alternative cyclisation pathways were possible and so could yield different molecular scaffolds. In the case of the α‐diazo amide **5**, (at least) two cyclisations were possible, leading to either the β‐lactam **6** (by insertion into an aryl C−H bond) or the oxindole **7** (by insertion into a benzylic C−H bond) (Figure 5, Panel A). As part of this project, we screened both dirhodium(II) catalysts and solvents, because it was not obvious at the outset which conditions would be both successful and selective with the functionalised substrate **5** (Panels B and C). We note, in retrospect, that the catalysts screened reside in all four quadrants of the catalyst map that is presented here. In this discovery workflow, the reactions had been performed in 96‐well plate format, and had been assembled from stock solutions (reaction volume: 100 μL; final concentrations: α‐diazo amide, 100 mm; catalyst, 1 mm); reaction outcomes were determined by HPLC.

We can now combine these screening results with our map of catalyst space. The outcome of the reaction varied widely as a function of catalyst (Figure 5, Panels B and C), with the yield of **6** ranging from 0–70 % across all catalysts and the three solvents (DCM, toluene and EtOAc). In terms of selectivity, the β‐lactam **6** was the major product with catalysts at positive PC2: **3 a** and **3 c** (which bear aliphatic carboxylates); **3 q** (Rh_2_esp_2_, which has a bridging dicarboxylate ligand); and **4 a** (with a cyclic carboxamidate *N*,*O* ligand). Conversion was generally good with these catalysts, and high yields of **6** were observed by HPLC. In contrast, the reaction had poor conversion with **4 d** and **4 m** which have *N*,*O* ligands based on cyclic ureas or oxazolidinones (and negative PC1 and PC2). The oxindole **7** was generally selectively formed, and formed in good HPLC yields, with **3 e** and **3 h** which bear perfluoroalkyl carboxylates (and have positive PC1 and negative PC2). Although not investigated in this study, we note that **4 z** (with trifluoroacetamidate ligands), which also has negative PC2, has been reported to induce selective cyclisation of related, but less functionalised, substrates to yield oxindoles.^[24]^ Based on this observation, and the descriptors which load highly on PC2, this shows that changes to catalyst Lewis acidity modulate the reaction selectivity more profoundly than the nature of the ligand donor atoms. Both carboxylate and carboxamidate ligands can lead to high yields of **6** in this reaction.

We note that our catalyst map could have guided catalyst selection in this discovery project,^[25]^ both to help identify promising catalyst classes, and to optimise selective syntheses of both **6** and **7** based on visualising the outcomes as shown. The most promising reactions were scaled up 50‐fold, enabling both products to be isolated in good yield: the β‐lactam agonist **6** (75 % yield with Rh_2_esp_2_ **3 q** in EtOAc) and the oxindole **7** (92 % yield with Rh_2_tfa_4_ **3 e** in CH_2_Cl_2_). In common with other studies,[^5^, ^25b^, ^25c^] we found that similar outcomes were observed on both scales, confirming that the analysis of microscale reactions can facilitate the optimisation of preparative dirhodium(II)‐catalysed reactions.

## Summary and Conclusions

We envisage that our catalyst map and the underlying database will facilitate the optimisation of novel Rh‐catalysed transformations. Crucially, our parameterisation was independent of mechanism and reaction mode, which may enable our database to support the exploration of the reactivity landscape for any process catalysed by dirhodium(II) complexes. We have shown that the alternative outcomes of the cyclisation of a functionalised substrate correlated with the catalyst location within the map, highlighting clusters of catalysts favouring different products. The map can assist the selection of catalyst screening sets for application in Design of Experiments (DoE) approaches to optimisation.[^7c^, ^17b^, ^26^] Such catalyst sets may have value in high‐throughput reaction optimisation within either a drug discovery or a process chemistry context. To enable identification of fertile regions for experimental investigation, catalyst sets could benefit from the addition of novel catalysts that complement currently‐available rhodium(II) complexes, that is, which occupy areas not sampled on the current maps. The catalyst map presented here may therefore also spur the development of catalysts with properties that complement those of currently available dirhodium(II) catalysts. We have shown that our database, capturing a diverse range of catalysts, could have supported the discovery of a series of androgen receptor agonists, and this approach may help more broadly to expand the reaction toolkit for molecular discovery.

## Computational Section

Optimised geometries for all rhodium(II) complexes were calculated with the Gaussian09 software package (see Supporting Information for full citation) in isolation using the standard BP86^[19]^ density functional as implemented in Gaussian with the DZP basis set 6‐31G(d)^[20]^ on all atoms apart from rhodium where the Stuttgart/Dresden effective core potential MWB28^[21]^ was used. Optimisations used “tight” convergence criteria. Vibrational frequencies were not computed, and so the energetic data do not include a correction for zero‐point energy, although we would expect this to be quite small. In the absence of frequency calculations, stationary points have not been verified as minima. However, most ligands and complexes are large and optimization to transition states seems unlikely for these carefully built low symmetry starting geometries. Geometry optimisations were started from crystal structure geometries of the complex of interest (see CSD refcodes in Table S3), or by careful structural modification of related complexes.

Full computational details have been given in the Supporting Information.

## Conflict of interest

The authors declare no conflict of interest.

## Supporting information

As a service to our authors and readers, this journal provides supporting information supplied by the authors. Such materials are peer reviewed and may be re‐organized for online delivery, but are not copy‐edited or typeset. Technical support issues arising from supporting information (other than missing files) should be addressed to the authors.

Supplementary

## References

[1a] J. Boström , D. G. Brown , R. J. Young , G. M. Keserü , Nat. Rev. Drug Disc. 2018, 17, 709–727; 10.1038/nrd.2018.116 30140018

[1b] D. C. Blakemore , L. Castro , I. Churcher , D. C. Rees , A. W. Thomas , D. M. Wilson , A. Wood , Nat. Chem. 2018, 10, 383–394; 29568051 10.1038/s41557-018-0021-z

[1c] S. D. Roughley , A. M. Jordan , J. Med. Chem. 2011, 54, 3451–3479; 21504168 10.1021/jm200187y

[1d] T. W. J. Cooper , I. B. Campbell , S. J. F. Macdonald , Angew. Chem. Int. Ed. 2010, 49, 8082–8091; 10.1002/anie.201002238 20859975

[2] J. S. Carey , D. Laffan , C. Thomson , M. T. Williams , Org. Biomol. Chem. 2006, 4, 2337–2347. 16763676 10.1039/b602413k

[3] D. G. Brown , J. Boström , J. Med. Chem. 2016, 59, 4443–4458. 26571338 10.1021/acs.jmedchem.5b01409

[4a] S. M. Mennen , C. Alhambra , C. L. Allen , M. Barberis , S. Berritt , T. A. Brandt , A. D. Campbell , J. Castañón , A. H. Cherney , M. Christensen , D. B. Damon , J. Eugenio de Diego , S. García-Cerrada , P. García-Losada , R. Haro , J. Janey , D. C. Leitch , L. Li , F. Liu , P. C. Lobben , D. W. C. MacMillan , J. Magano , E. McInturff , S. Monfette , R. J. Post , D. Schultz , B. J. Sitter , J. M. Stevens , I. I. Strambeanu , J. Twilton , K. Wang , M. A. Zajac , Org. Process Res. Dev. 2019, 23, 1213–1242;

[4b] S. D. Dreher , React. Chem. Eng. 2019, 4, 1530–1535;

[4c] S. W. Krska , D. A. DiRocco , S. D. Dreher , M. Shevlin , Acc. Chem. Res. 2017, 50, 2976–2985. 29172435 10.1021/acs.accounts.7b00428

[5a] D. Perera , J. W. Tucker , S. Brahmbhatt , C. J. Helal , A. Chong , W. Farrell , P. Richardson , N. W. Sach , Science 2018, 359, 429; 29371464 10.1126/science.aap9112

[5b] N. J. Gesmundo , B. Sauvagnat , P. J. Curran , M. P. Richards , C. L. Andrews , P. J. Dandliker , T. Cernak , Nature 2018, 557, 228–232; 29686415 10.1038/s41586-018-0056-8

[5c] T. Cernak , N. J. Gesmundo , K. Dykstra , Y. Yu , Z. Wu , Z.-C. Shi , P. Vachal , D. Sperbeck , S. He , B. A. Murphy , L. Sonatore , S. Williams , M. Madeira , A. Verras , M. Reiter , C. H. Lee , J. Cuff , E. C. Sherer , J. Kuethe , S. Goble , N. Perrotto , S. Pinto , D.-M. Shen , R. Nargund , J. Balkovec , R. J. DeVita , S. D. Dreher , J. Med. Chem. 2017, 60, 3594–3605; 28252959 10.1021/acs.jmedchem.6b01543

[5d] A. Buitrago Santanilla , E. L. Regalado , T. Pereira , M. Shevlin , K. Bateman , L.-C. Campeau , J. Schneeweis , S. Berritt , Z.-C. Shi , P. Nantermet , Y. Liu , R. Helmy , C. J. Welch , P. Vachal , I. W. Davies , T. Cernak , S. D. Dreher , Science 2015, 347, 49. 25554781 10.1126/science.1259203

[6] D. J. Durand , N. Fey , Chem. Rev. 2019, 119, 6561–6594. 30802036 10.1021/acs.chemrev.8b00588

[7a] N. Fey , Chem. Cent. J. 2015, 9, 38; 26113874 10.1186/s13065-015-0104-5 PMC4480443

[7b] A. Ekebergh , C. Lingblom , P. Sandin , C. Wennerås , J. Mårtensson , Org. Biomol. Chem. 2015, 13, 3382–3392; 25658776 10.1039/c4ob02694b

[7c] J. D. Moseley , P. M. Murray , J. Chem. Technol. Biotechnol. 2014, 89, 623–632.

[8] A. Ford , H. Miel , A. Ring , C. N. Slattery , A. R. Maguire , M. A. McKervey , Chem. Rev. 2015, 115, 9981–10080. 26284754 10.1021/acs.chemrev.5b00121

[9a] G. Karageorgis , M. Dow , A. Aimon , S. Warriner , A. Nelson , Angew. Chem. Int. Ed. 2015, 54, 13538–13544; 10.1002/anie.201506944 PMC4648041 26358926

[9b] G. Karageorgis , S. Warriner , A. Nelson , Nat. Chem. 2014, 6, 872–876; 25242481 10.1038/nchem.2034

[9c] A. Green , F. Hobor , C. Tinworth , S. Warriner , A. Wilson , A. Nelson , Chem. Eur. J. 2020, 26, 10682–10689. 32458465 10.1002/chem.202002153 PMC7496268

[10a] B. Wei , J. C. Sharland , P. Lin , S. M. Wilkerson-Hill , F. A. Fullilove , S. McKinnon , D. G. Blackmond , H. M. L. Davies , ACS Catal. 2020, 10, 1161–1170;

[10b] B. D. McLarney , S. Hanna , D. G. Musaev , S. France , ACS Catal. 2019, 9, 4526–4538;

[10c] H. M. L. Davies , J. Org. Chem. 2019, 84, 12722–12745; 31525891 10.1021/acs.joc.9b02428 PMC7232105

[10d] K. Liao , Y.-F. Yang , Y. Li , J. N. Sanders , K. N. Houk , D. G. Musaev , H. M. L. Davies , Nat. Chem. 2018, 10, 1048–1055; 30082883 10.1038/s41557-018-0087-7 PMC6650386

[10e] K. Liao , W. Liu , Z. L. Niemeyer , Z. Ren , J. Bacsa , D. G. Musaev , M. S. Sigman , H. M. L. Davies , ACS Catal. 2018, 8, 678–682;

[10f] E. N. Bess , D. M. Guptill , H. M. L. Davies , M. S. Sigman , Chem. Sci. 2015, 6, 3057–3062; 29403640 10.1039/c5sc00357a PMC5763982

[10g] D. Gillingham , N. Fei , Chem. Soc. Rev. 2013, 42, 4918–4931; 23407887 10.1039/c3cs35496b

[10h] H. M. L. Davies , D. Morton , Chem. Soc. Rev. 2011, 40, 1857–1869; 21359404 10.1039/c0cs00217h

[10i] A. Padwa , M. D. Weingarten , Chem. Rev. 1996, 96, 223–270; 11848752 10.1021/cr950022h

[10j] A. Padwa , D. J. Austin , A. T. Price , M. A. Semones , M. P. Doyle , M. N. Protopopova , W. R. Winchester , A. Tran , J. Am. Chem. Soc. 1993, 115, 8669–8680.

[11a] D. Balcells , A. Nova , ACS Catal. 2018, 8, 3499–3515;

[11b] S. R. Hare , D. J. Tantillo , Chem. Sci. 2017, 8, 1442–1449; 28451284 10.1039/c6sc03745c PMC5390789

[11c] C. L. McMullin , N. Fey , J. N. Harvey , Dalton Trans. 2014, 43, 13545–13556; 25091386 10.1039/c4dt01758g

[11d] L. Ye , Y. Wang , D. H. Aue , L. Zhang , J. Am. Chem. Soc. 2012, 134, 31–34. 22176593 10.1021/ja2091992 PMC3257394

[12a] Z. Liu , C. Patel , J. N. Harvey , R. B. Sunoj , Phys. Chem. Chem. Phys. 2017, 19, 30647–30657; 29116284 10.1039/c7cp06508f

[12b] Q. Peng , F. Duarte , R. S. Paton , Chem. Soc. Rev. 2016, 45, 6093–6107; 27722685 10.1039/c6cs00573j

[12c] A. F. Zahrt , S. V. Athavale , S. E. Denmark , Chem. Rev. 2020, 120, 1620–1689. 31886649 10.1021/acs.chemrev.9b00425 PMC7018559

[13] J. Jover , N. Fey , Chem. Asian J. 2014, 9, 1714–1723. 24668590 10.1002/asia.201301696

[14] A. R. Rosales , T. R. Quinn , J. Wahlers , A. Tomberg , X. Zhang , P. Helquist , O. Wiest , P.-O. Norrby , Chem. Commun 2018, 54, 8294–8311. 10.1039/c8cc03695k PMC6069518 29971313

[15] G. Occhipinti , H.-R. Bjørsvik , V. R. Jensen , J. Am. Chem. Soc. 2006, 128, 6952–6964. 16719476 10.1021/ja060832i

[16a] M. C. Pirrung , H. Liu , A. T. Morehead , J. Am. Chem. Soc. 2002, 124, 1014–1023; 11829610 10.1021/ja011599l

[16b] C. Werlé , R. Goddard , P. Philipps , C. Farès , A. Fürstner , J. Am. Chem. Soc. 2016, 138, 3797–3805. 26910883 10.1021/jacs.5b13321

[17a] N. Fey , A. C. Tsipis , S. E. Harris , J. N. Harvey , A. G. Orpen , R. A. Mansson , Chem. Eur. J. 2006, 12, 291–302; 10.1002/chem.200500891 16278917

[17b] J. Jover , N. Fey , J. N. Harvey , G. C. Lloyd-Jones , A. G. Orpen , G. J. J. Owen-Smith , P. Murray , D. R. J. Hose , R. Osborne , M. Purdie , Organometallics 2010, 29, 6245–6258. 10.1021/om300312t PMC4034078 24882917

[18a] N. Fey , J. N. Harvey , G. C. Lloyd-Jones , P. Murray , A. G. Orpen , R. Osborne , M. Purdie , Organometallics 2008, 27, 1372–1383; 10.1021/om300312t PMC4034078 24882917

[18b] J. Jover , N. Fey , J. N. Harvey , G. C. Lloyd-Jones , A. G. Orpen , G. J. J. Owen-Smith , P. Murray , D. R. J. Hose , R. Osborne , M. Purdie , Organometallics 2012, 31, 5302–5306. 24882917 10.1021/om300312t PMC4034078

[19a] A. D. Becke , Phys. Rev. A 1988, 38, 3098–3100; 10.1103/physreva.38.3098 9900728

[19b] J. P. Perdew , Phys. Rev. B 1986, 33, 8822–8824. 10.1103/physrevb.33.8822 9938299

[20a] M. J. Frisch , J. A. Pople , J. S. Binkley , J. Chem. Phys. 1984, 80, 3265–3269;

[20b] P. C. Hariharan , J. A. Pople , Theor. Chem. Acta 1973, 28, 213–222.

[21] D. Andrae , U. Häußermann , M. Dolg , H. Stoll , H. Preuß , Theor. Chem. Acta 1990, 77, 123–141.

[22a] S. Aguado-Ullate , S. Saureu , L. Guasch , J. J. Carbó , Chem. Eur. J. 2012, 18, 995–1005; 22190342 10.1002/chem.201101230

[22b] S. Aguado-Ullate , M. Urbano-Cuadrado , I. Villalba , E. Pires , J. I. García , C. Bo , J. J. Carbó , Chem. Eur. J. 2012, 18, 14026–14036. 22987760 10.1002/chem.201201135

[23] T. Hastie , R. Tibshirani , J. Friedman , The Elements of Statistical Learning, 2nd Ed. , Springer , New York , 2009.

[24a] D. S. Brown , M. C. Elliott , C. J. Moody , T. J. Mowlem , J. P. Marino Jr , A. Padwa , J. Org. Chem. 1994, 59, 2447–2455;

[24b] S. Miah , A. M. Z. Slawin , C. J. Moody , S. M. Sheehan , J. P. Marino , M. A. Semones , A. Padwa , I. C. Richards , Tetrahedron 1996, 52, 2489–2514.

[25a] P. M. Murray , F. Bellany , L. Benhamou , D.-K. Bučar , A. B. Tabor , T. D. Sheppard , Org. Biomol. Chem. 2016, 14, 2373–2384; 26699438 10.1039/c5ob01892g

[25b] A. McNally , C. K. Prier , D. W. C. MacMillan , Science 2011, 334, 1114–1117; 22116882 10.1126/science.1213920 PMC3266580

[25c] M. Shevlin , ACS Med. Chem. Lett. 2017, 8, 601–607. 28626518 10.1021/acsmedchemlett.7b00165 PMC5467193

[26] P. M. Murray , S. N. G. Tyler , J. D. Moseley , Org. Process Res. Dev. Org. Proc. Res. Dev. 2013, 17, 40–46.

