# Supplementary material for: Computational Mapping of Dirhodium(II) Catalysts
Source: Chemistry. 2021 Jan 12;27(7):2402–9. doi: 10.1002/chem.202003801 (PMC7898874; doi:10.1002/chem.202003801)
Supplement: Supplementary file 1 — Supplementary [file CHEM-27-2402-s001.pdf]

Chemistry–A European Journal

Supporting Information

Computational Mapping of Dirhodium(II) Catalysts

Adam I. Green,[a] Christopher P. Tinworth,[b] Stuart Warriner,[a] Adam Nelson,*[a] and Natalie Fey*[c]

Author Contributions

N.F. Conceptualization: Equal; Data curation: Supporting; Formal analysis: Supporting; Methodology: Equal; Supervision: Supporting; Writing - Original Draft: Supporting; Writing - Review & Editing: Lead
A.G. Data curation: Lead; Formal analysis: Lead; Investigation: Lead; Writing - Original Draft: Supporting
C.T. Data curation: Supporting; Supervision: Supporting
S.W. Supervision: Supporting
A.N. Conceptualization: Equal; Formal analysis: Supporting; Funding acquisition: Lead; Methodology: Equal; Project administration: Lead; Supervision: Lead; Writing - Original Draft: Equal; Writing - Review & Editing: Supporting.

Table of Contents

Full Computational Details 2
Design of Descriptor Database 4
Modelled Rhodium(II) complexes 11
Correlation Analysis of Calculated Descriptors 14
Principal Component Analysis of Database 15
Relationship with Experimental Data 26
References 29

Full Computational Details

Optimised geometries for all rhodium(II) complexes were calculated with the Gaussian09[1] software package in isolation using the standard BP86[2–4] density functional as implemented in Gaussian with the DZP basis set 6-31G(d)[5–8] on all atoms apart from rhodium where the Stuttgart/Dresden effective core potential MWB28[9] was used. Optimisations used 'tight' convergence criteria. Vibrational frequencies were not computed, and so the energetic data do not include a correction for zero-point energy, although we would expect this to be quite small. In the absence of frequency calculations, stationary points have not been verified as minima. However, most ligands and complexes are large and optimization to transition states seems unlikely for these carefully built low symmetry starting geometries.

Geometry optimisations were started from crystal structure geometries of the complex of interest (see CSD refcodes in Table S3 below), or by careful structural modification of related complexes.

Conformational searches used the default MMX force field in PCModel.[10] GMMX was used for stochastic conformational searches, generally with default settings. 500 iteration conformational searches were performed on the dirhodium complex as well as its carbene complex (stop criteria defined as Emin found 10 times and duplicates found 50 times). These conformational searchers were attempted for a subset of complexes to enhance conformational sampling but were hampered by missing parameters (circumvented by replacing atoms with elements where parameters existed and suitable restraints) and difficulties with convergence, most likely due to the large number of connected rings arising from the dirhodium core. A selection of conformers were then re-optimised fully at the DFT level, as described above. A full re-parameterisation of the force field lay outside the scope of this project, but for **3d** and **4g**, we explored the impact of conformational change on the descriptors. These are summarised in Tables S1 and S2 below. While for **3d**, the crystal structure geometry led to the lowest energy conformer, this was not the case for **4g**. However, the range of energies found for **4g** was small and the descriptors for the XRD-derived conformer and the lowest energy species found are reasonably similar. Inspection of descriptors shows limited variation in structural and energetic parameters, but a larger range for the steric descriptors (He_8 and |wV|) as well as for the energy for Diazo precursor to form the carbene complex (ΔE_{coord}), as might be expected.[11] While we recognise that conformational change will have a larger impact for the prediction of selectivity, reactivity and dynamic behaviour of some of the catalysts, in view of the computational problems with sampling conformer space reliably, we decided against including Boltzmann-averaged descriptors in the present version of this database.

Table S1. TBSP **3d** conformers

Parameter/Complex	TSBP (XRD)	TBSP_m1_ c1	TBSP_m1b_ c1	TBSP_m1b_ c5	TBSP_m1b_ c10	range	TSBP_carb (XRD)	TBSP_carb_ c1	TBSP_carb_ c3	range
Rel. E, kcal/mol	0.00	3.78	3.74	4.35	2.73	4.35	0.00	10.00	8.12	10.00
r(Rh-Rh), 1	2.392	2.400	2.400	2.394	2.392	0.008	-	-	-	-
r(Rh-L), 1	2.058	2.059	2.060	2.057	2.057	0.003	-	-	-	-
∡(Rh-Rh-L), 1	88.6	88.5	88.5	88.6	88.6	0.1	-	-	-	-
∡(O-C-X)	126.5	126.5	126.5	126.5	126.5	0.0	-	-	-	-
r(αC-R1)	1.537	1.538	1.538	1.537	1.537	0.001	-	-	-	-
r(Rh-Rh), 2	-	-	-	-	-	-	2.466	2.464	2.464	0.002
r(Rh-L), 2	-	-	-	-	-	-	2.069	2.069	2.069	0.000
∡(Rh-Rh-L), 2	-	-	-	-	-	-	87.7	87.7	87.7	0.0
r(Rh-C)	-	-	-	-	-	-	1.979	1.975	1.978	0.004
∡(C-C-C)	-	-	-	-	-	-	113.8	114.4	115.6	1.7
ΔE(coord)	-	-	-	-	-	-	22.67	12.66	14.55	10.00
He8	-	-	-	-	-	-	33.25	37.49	33.19	4.29
|wV|	-	-	-	-	-	-	26.70	26.70	19.40	7.30
HOMO, 1	-0.1665	-0.1699	-0.1696	-0.1701	-0.1695	0.0036	-	-	-	-
LUMO, 1	-0.1310	-0.1304	-0.1302	-0.1347	-0.1340	0.0045	-	-	-	-
ΔE(FMO)	-0.0355	-0.0395	-0.0394	-0.0354	-0.0355	0.0040	-	-	-	-
Q Rh, 1	0.8072	0.8164	1.0406	1.1353	1.1318	0.3281	-	-	-	-
Q(Donor Atoms, mean), 1	-4.2841	-4.2822	-4.3875	-4.4222	-4.4212	0.1400	-	-	-	-
Q(L, mean), 1	-1.0253	-1.1395	-1.0406	-1.1353	-1.1319	0.1142	-	-	-	-

Table S2. MEPY **4g** conformers

Parameter/Complex	MEPY	MEPY_c1	MEPY_c42	range	MEPY_carb (XRD)	MEPY_carb_c1	MEPY_carb_c35	MEPY_carb_c75	range
Rel. E, kcal/mol	1.50	0.00	5.40	5.40	1.68	0.23	0.50	0.00	1.68
r(Rh-Rh), 1	2.466	2.463	2.463	0.004	-	-	-	-	-
r(Rh-L), 1	2.064	2.068	2.061	0.006	-	-	-	-	-
∡(Rh-Rh-L), 1	87.8	88.0	88.1	0.2	-	-	-	-	-
∡(O-C-X)	125.6	125.6	125.6	0.0	-	-	-	-	-
r(αC-R1)	1.527	1.525	1.524	0.002	-	-	-	-	-
r(Rh-Rh), 2	-	-	-	-	2.540	2.522	2.536	2.533	0.017
r(Rh-L), 2	-	-	-	-	2.070	2.072	2.071	2.071	0.002
∡(Rh-Rh-L), 2	-	-	-	-	87.0	87.2	87.1	87.0	0.2
r(Rh-C)	-	-	-	-	1.992	1.974	1.996	1.994	0.022
∡(C-C-C)	-	-	-	-	110.4	110.7	111.6	110.3	1.3
ΔE(coord)	-	-	-	-	8.73	10.18	9.91	10.41	1.68
He8	-	-	-	-	58.71	76.19	54.45	60.84	21.74
|wV|	-	-	-	-	14.70	19.80	18.00	13.70	6.10
HOMO, 1	-0.1261	-0.1337	-0.1330	0.0076	-	-	-	-	-
LUMO, 1	-0.0866	-0.1175	-0.1088	0.0309	-	-	-	-	-
ΔE(FMO)	-0.0396	-0.0162	-0.0242	0.0233	-	-	-	-	-
Q Rh, 1	0.6234	0.6197	0.6106	0.0128	-	-	-	-	-
Q(Donor Atoms, mean), 1	-4.1089	-4.1169	-4.1078	0.0091	-	-	-	-	-
Q(L, mean), 1	-0.8028	-0.7340	-0.7828	0.0687	-	-	-	-	-

Design of Descriptor Database

The 14 steric and electronic quantum chemical descriptors outlined below (Table **S4**) were then captured from optimised geometries. The coordination energy of the carbene generated from a symmetrical α-diazo malonamide precursor was calculated from converged energies (a.u.) using the equation below.

$$\varDelta E(coord)\ kcal\ mol^{-1}\ =\ 627.5095\times((E\mathbf{1}\ +E\boldsymbol{Diazo})-(E\mathbf{2}+\ E\boldsymbol{N_2}))$$

Natural population analysis (NBO) was calculated using the converged complex and the energy difference between the HOMO and LUMO molecular orbitals was calculated in atomic units using the equation below.

$$\varDelta E(FMO)\ a.u.=\ LUMO-HOMO$$

Other parameters captured from the NBO analysis include the charge on the rhodium atoms, charge on the ligand donor atoms and the average charge on the ligands.

For the PCA model 48 ligands (Table **S3**) were optimised as complexes **1** and **2** (n = 96) and 19 chemical descriptors were extracted from these optimised complex geometries. Each descriptor (x_i) was scaled to dimensionless values (x) using the equation below.

$$x = \frac{(xi - u)}{s}$$

Where $u = \frac{1}{N}\sum_{i=1}^{N} xi$ (sample mean of x), N = number of data points

$s = \sqrt{\frac{1}{N}\sum_{i=1}^{N}(xi - u)^2}$ (sample standard deviation of x).

Chemical descriptors considered:

1. All bond lengths and angles (including dihedral angles) for both complexes **1** and **2** calculated at BP86/6-31G(d)/MWB28 level of theory in Gaussian09. Lengths and angles were assessed for their response to carbene formation and the following descriptors were selected: r(Rh-Rh), r(Rh-L), ∡(Rh-Rh-L), r(αC-R^1), ∡(O-C-X), r(Rh-C) and ∡(C-C-C) as these showed a clear response to changes in ligand properties, as well as being computationally robust.
2. Binding energies of ancillary ligands, including diazo malonamide (BP86/6-31G(d)/MWB28), methyl 2-diazo-2-phenylacetate (BP86/6-31G(d)/MWB28), and acetonitrile (B3LYP/6-31G(d)/MWB28). Complexes bearing acetonitrile ligands were found to possess extreme bond angles for complexes with carboxamidate ligands and were thus removed from subsequent analyses (Figure S3). Acetonitrile complexes were initially calculated using the B3LYP functional, however, due to an inability to obtain convergence on several carboxamidate complexes, the functional was changed to BP86 for all other calculations. This functional may over-bind slightly but is computationally robust and converges reliably for all catalysts considered here.
3. Several steric descriptors were considered, including an adapted version of the He_8 ring used in Bristol's LKBs,[12] Distance-Weighted Volume[13] and first-generation Sterimol parameters.[14] The interaction energies between the dirhodium complexes and a ring of 8 helium atoms, i.e. He_8 ring interaction energies were calculated as single-point energies at BP86/6-31G(d)/MWB28 level of theory, where the He_8 ring was aligned 1.9 Å from the rhodium core (average r(Rh-C) bond length). Distance-Weighted Volume was derived from the MolQuO web app (http://rodi.urv.es/~carbo/quadrants/index.html), aligning the quadrants with the Rh-C bond and then removing the carbene ligand from the optimised geometry (Figure S2). First-generation Sterimol parameters were calculated by aligning the L vector with the Rh-Rh bond and using a python script available here: https://github.com/bobbypaton/Sterimol. Sterimol

parameters were found to be prone to outliers due to the extreme size of many of the ligands and less capable of describing the steric environment around the Rh atoms, so were not used in subsequent analysis. We note that the more recent versions of this descriptor, which explore conformational variation (wSterimol),[15] may be able to address this, provided conformational searches can be carried out (alas, see above).

4. Descriptors derived from the Natural Population Analysis of the converged complexes including: the HOMO and LUMO energies for complexes **1** and **2** (Figures S3 – S5); charges on atoms including the core Rhodium atoms, ligand donor atoms and the average charge on the ligand for complexes **1** and **2**; and the difference in energy between the frontier molecular orbitals.
5. We also considered calculating free ligand descriptors to supplement this database, aligning more fully with Bristol's Ligand Knowledge Bases. However, existing LKBs focus on a single ligand and the effect of modifying this on both metal fragment and ligand coordination. In the present case, this is not particularly helpful, as we are interested in the effect of four ligands on the dirhodium "core". In addition, the ligands together determine the coordination site on Rh, so a ligand-centric "view" of this would be misleading. Finally, some ligands are capable of multiple coordination modes and, once metal coordination is removed, conformational space might lead to considerable variation which is not meaningful for the catalysis here. This does not preclude capturing these ligands, e.g. in the context of the recent LKB-bid database capturing wider bidentate ligand space, but we have not included such descriptors here.[16]

The descriptors used for analysis are included as Table 1 in the manuscript. Descriptors tested can also be found in a data table supplied separately as part of the ESI.

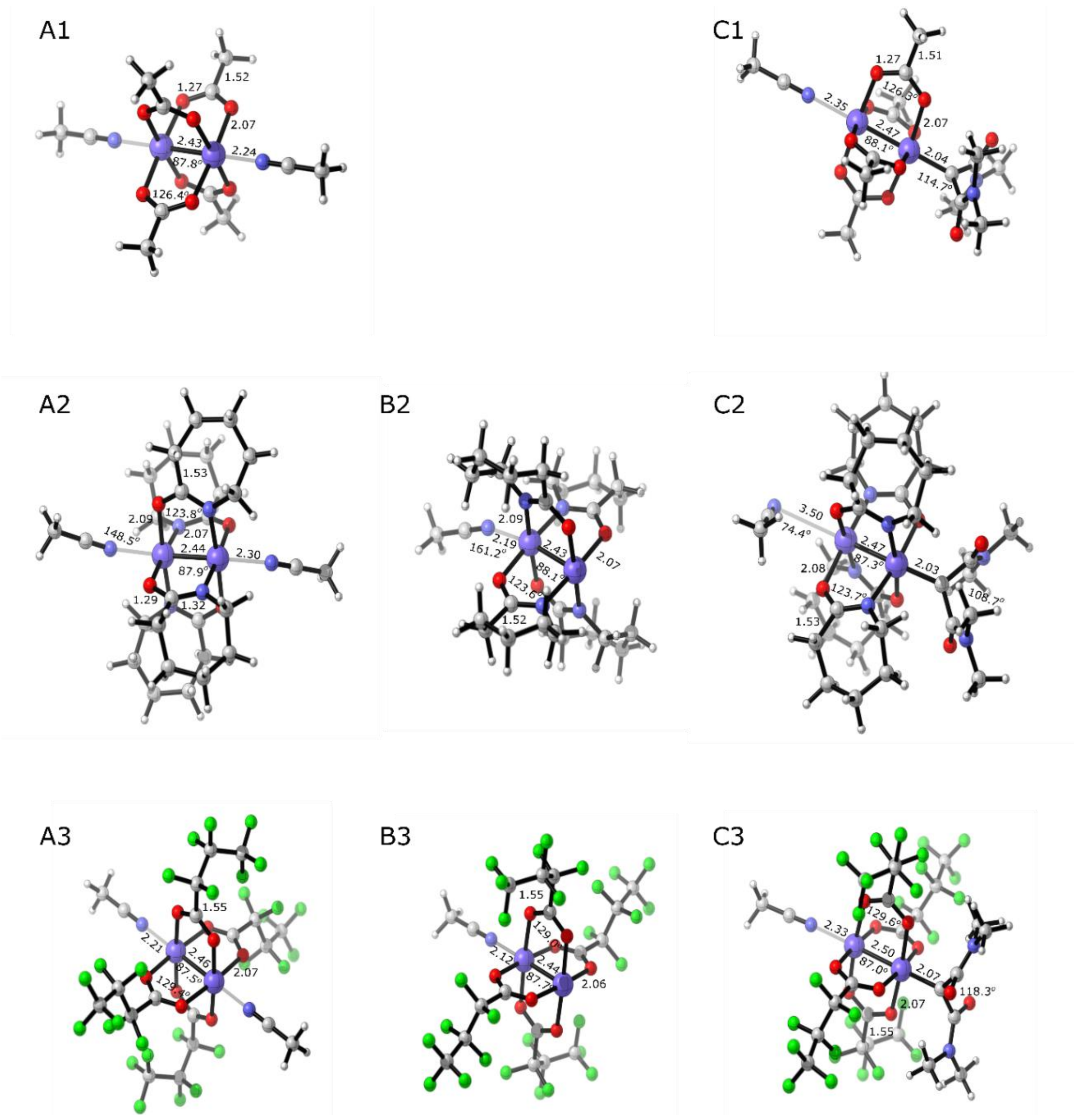

Figure S1. Example acetonitrile adducts calculated at B3LYP/6-31G(d)/MWB28 level of theory. A1: complex **3a** and 2 acetonitrile ligands. C1: complex **3a** with a carbene ligand and an acetonitrile ligand. A2: complex **4a** with 2 acetonitrile ligands. B2: complex **4a** with 1 acetonitrile ligand. C2: complex **4a** with a carbene ligand and an acetonitrile ligand. A3: complex **3h** with 2 acetonitrile ligands. B3: complex **3h** with 1 acetonitrile ligand. C3: complex **3h** with a carbene ligand and an acetonitrile ligand.

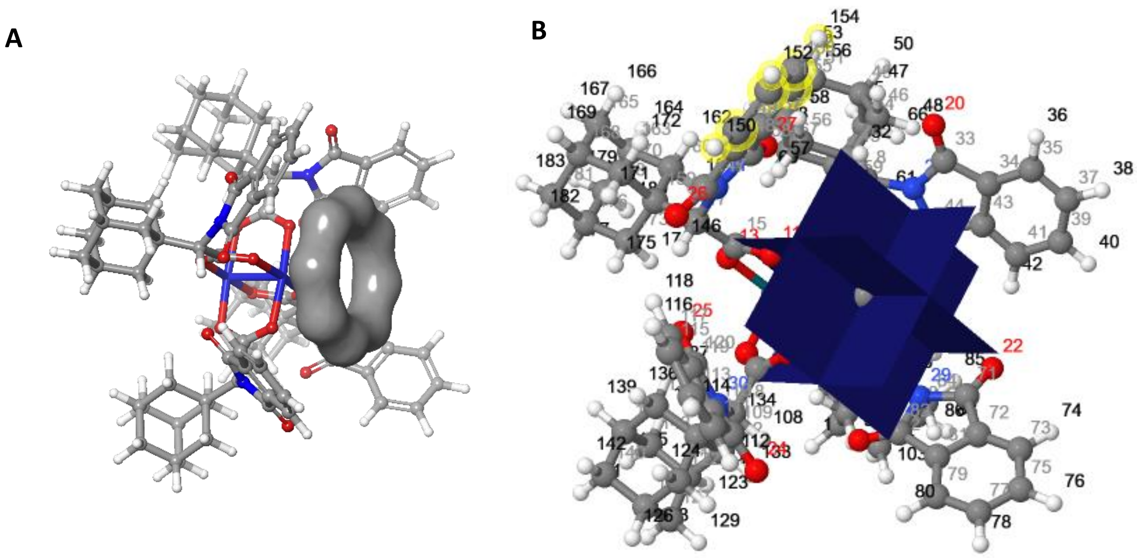

Figure S2. Exemplar steric parameters with complex **3s**. A: He_8 ring interaction energy calculated at a single point using BP86/6-31G(d)/MWB28 level of theory. B: |wV| derived from the MolQuO JSmol app.

Distance weighted volume, or quadrant occupation (see discussion above for details), gives a measure of the steric bulkiness of the ligand and its influence over the metal centre, and was calculated using the formula below.

$V_{W,k,l} = \sum_{i-1}^{n} \frac{r_i^k}{d_i^l}$ where k = 3 and l = 1, d^l = distance of atom to metal centre and r^k = van der Waals radius of atom.

For these dirhodium complexes, the frontier molecular orbitals correspond to metal orbitals available for bonding of additional ligands (e.g. the carbenes considered for descriptors) with the dirhodium core. This is illustrated in Figures S3-S5 below.

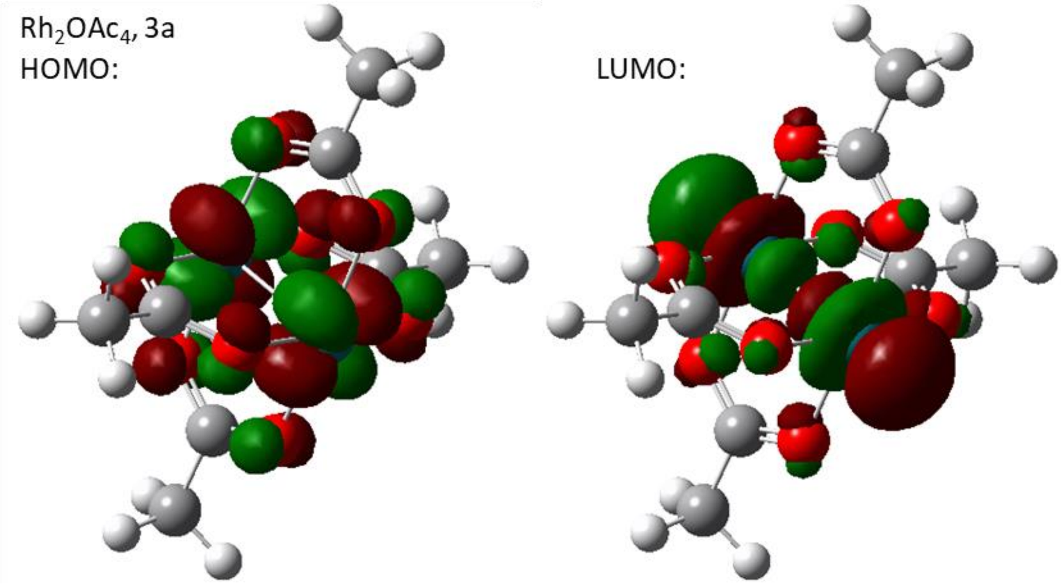

Figure S3. HOMO and LUMO for Rh_2OAc_4

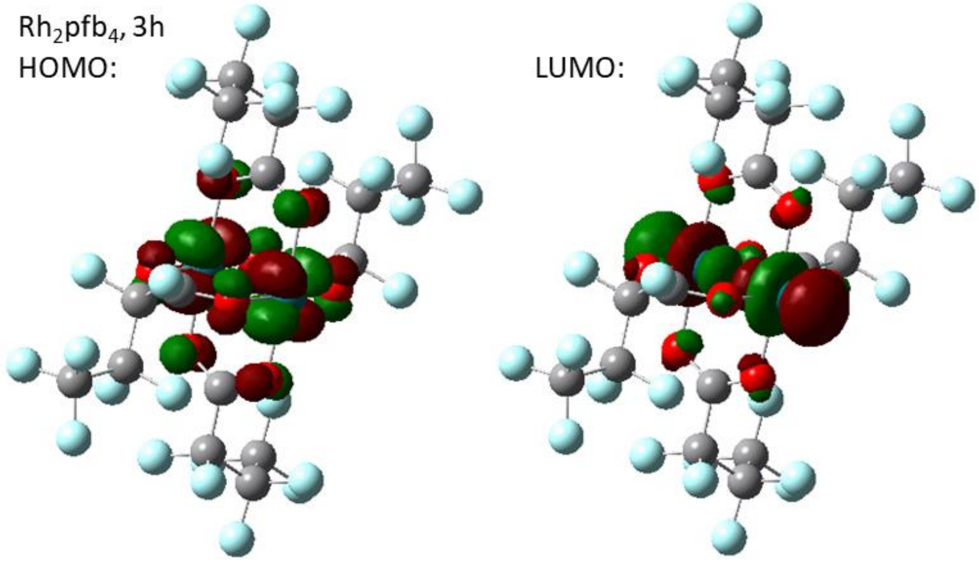

Figure S4. HOMO and LUMO for Rh_2pfb_4

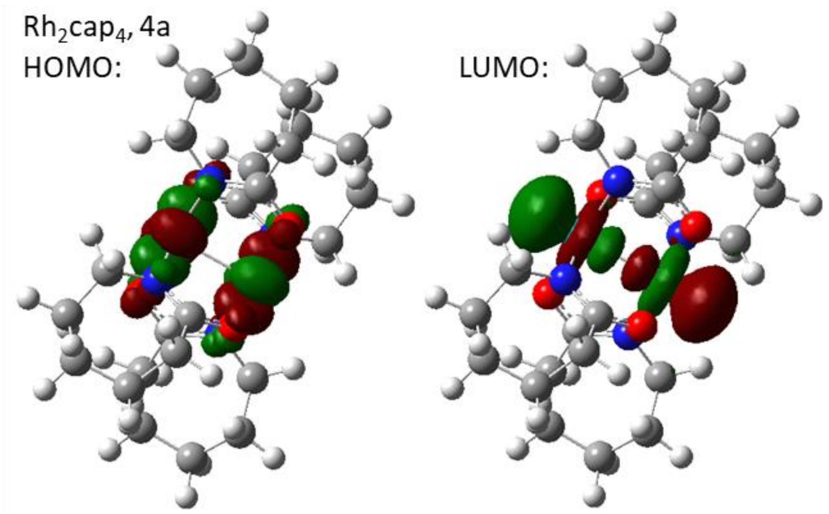

Figure S5. HOMO and LUMO for Rh_2cap_4

Modelled Rhodium(II) complexes

Table S3. All complexes calculated by DFT and featured in the PCA model. Where complexes were built from X-ray crystal structures the relevant CCDC code is given.

No.	Catalyst	Structure
3a	Rh_2OAc_4<br>BARPIT	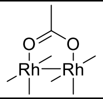
3b	Rh_2piv_4<br>HUWKOB	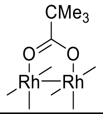
3c	Rh_2oct_4<br>XUZNOW	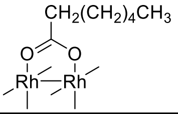
3d	Rh_2TBSP_4<br>LOPKOQ	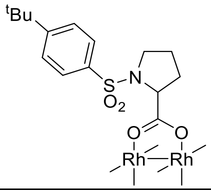
3e	Rh_2tfa_4<br>SAXYAS	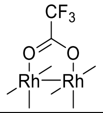
3f	Rh_2dfa_4	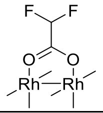
3g	Rh_2mfa_4	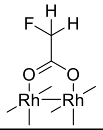
3h	Rh_2pfb_4<br>IGAFUR	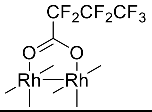
3i	Rh_2FPIV_4	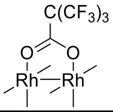
3j	Rh_2bnz_4	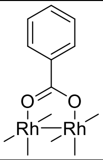
3k	Rh_2DABN_4	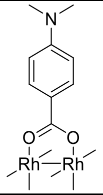
3l	Rh_2POMB_4	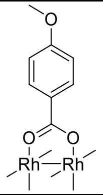
3m	Rh_2FBNZ_4	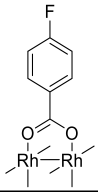
3n	Rh_2TFBN_4	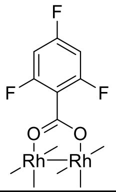
3o	Rh_2PFBN_4	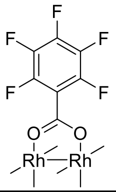
3p	Rh_2TFMB_4	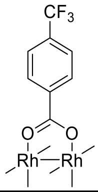
3q	Rh_2esp_2<br>KUTXAA	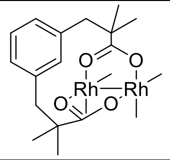
3r	Rh_2DOSP_4	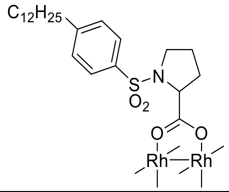
3s	Rh_2PTAD_4<br>WAJBAN	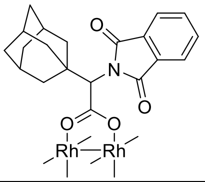
3t	Rh_2PTTL_4	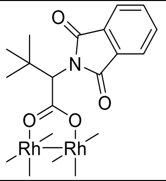
3u	Rh_2tPTTL_4	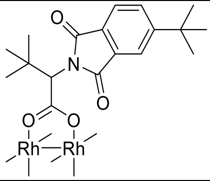

4a	Rh_2cap_4 QEZMOY	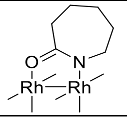
4b	Rh_2pip_4	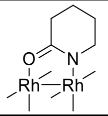
4c	Rh_2MPDO_4	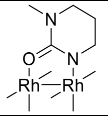
4d	Rh_2MEOX_4 PIZBAC	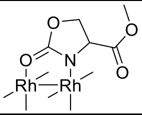
4e	Rh_2pyr_4	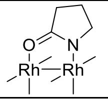
4f	Rh_2PYOH_4	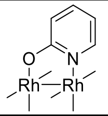
4g	Rh_2MEPY_4 HAWVAC	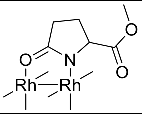
4h	Rh_2OXAZ_4	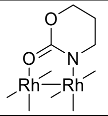
4i	Rh_2dhpo_4	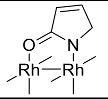
4j	-	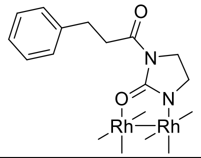
4k	Rh_2OXAL_4	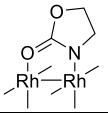
4l	Rh_2pyo_4	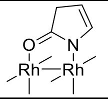
4m	Rh_2MPPIM_4	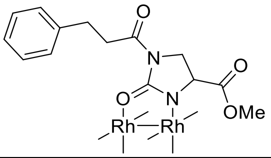
4n	Rh_2OXPN_4	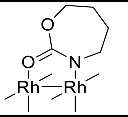
4o	Rh_2dhpd_4	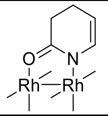

4p	-	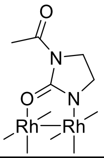
4q	Rh_2MIDO_4	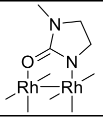
4r	Rh_2dhpy_4	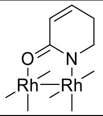
4s	Rh_2MACIM_4	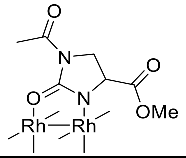
4t	Rh_2dfpyr_4	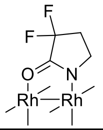
4u	Rh_2dfpip_4	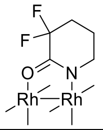
4v	Rh_2DIZO_4	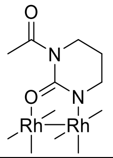
4w	Rh_2mfpyr_4	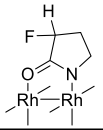
4x	Rh_2mfpip_4	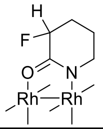
4y	Rh_2acam_4	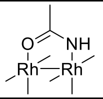
4z	Rh_2tfam_4	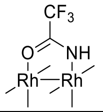
4aa	Rh_2DMU_4	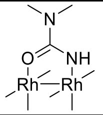

Table S4. Descriptors calculated. (Data for each complex can be accessed as a .csv file, part of the ESI)

Descriptor	Derivation	Diagram	Median Value	Range
r(Rh-Rh)	Rh-Rh bond length (Å), **1** and **2**	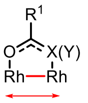	**1**: 2.409<br>**2**: 2.478	0.09
r(Rh-L)	Average Rh-Ligand bond length, **1** and **2**	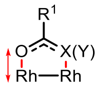	**1**: 2.057<br>**2**: 2.068	0.02
∡(Rh-Rh-L)	Average Rh-Rh-Ligand bond angle, **1** and **2**	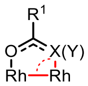	**1**: 88.6<br>**2**: 87.5	1.29
∡(O-C-X)	Average ligand bite angle, **1**	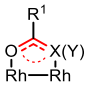	126.0	7.3
∡(C-C-C)	Carbene C-C-C angle, **2**	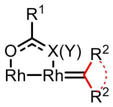	112.3	12.0
r(Rh-C)	Rhodium-Carbene bond length (Å), **2**	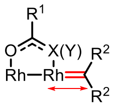	2.068	0.058
r(αC-R^1)	Average αC-Ligand Backbone (R^1) bond length (Å), **1**	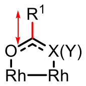	1.521	0.187
HOMO, **1**	Energy of HOMO for complex **1** (a.u.)	ESI Fig. S3 – S5	-0.1548	0.1159
LUMO, **1**	Energy of LUMO for complex **1** (a.u.)	ESI Fig. S3 – S5	-0.1299	0.0995
Q Rh, **1**	Charge on Rhodium atoms	-	0.6576	0.2988
Q(L, mean), **1**	Mean charge on ligands	-	-0.8457	0.5511
Q(Donor Atoms, mean), **1**	Mean charge on ligand donor atoms	-	-4.2696	1.2983
ΔE(FMO)	ΔE between HOMO and LUMO (a.u.)	-	0.0299	0.0324
|wV|	Distance-Weighted Volume,[19] **1**	$V_{W,k,l} = \sum_{i-1}^{n} \frac{r_i^k}{d_i^l}$	9.35	40.20
He_8	Interaction energy for **1** and ring of 8 Helium atoms[16] [kcal mol^{-1}]	Fig. 2<br>He_8 = E(He_8.[Rh-Rh])) – E(He_8) – E([Rh-Rh]))	35.33	47.75
ΔE(coord)	Energy for Diazo precursor to form the carbene complex [kcal mol^{-1}], **2**	ΔE(coord) = (E**1** + E**Diazo**) – (E**2** + EN_2)	19.75	24.09

Correlation Analysis of Calculated Descriptors

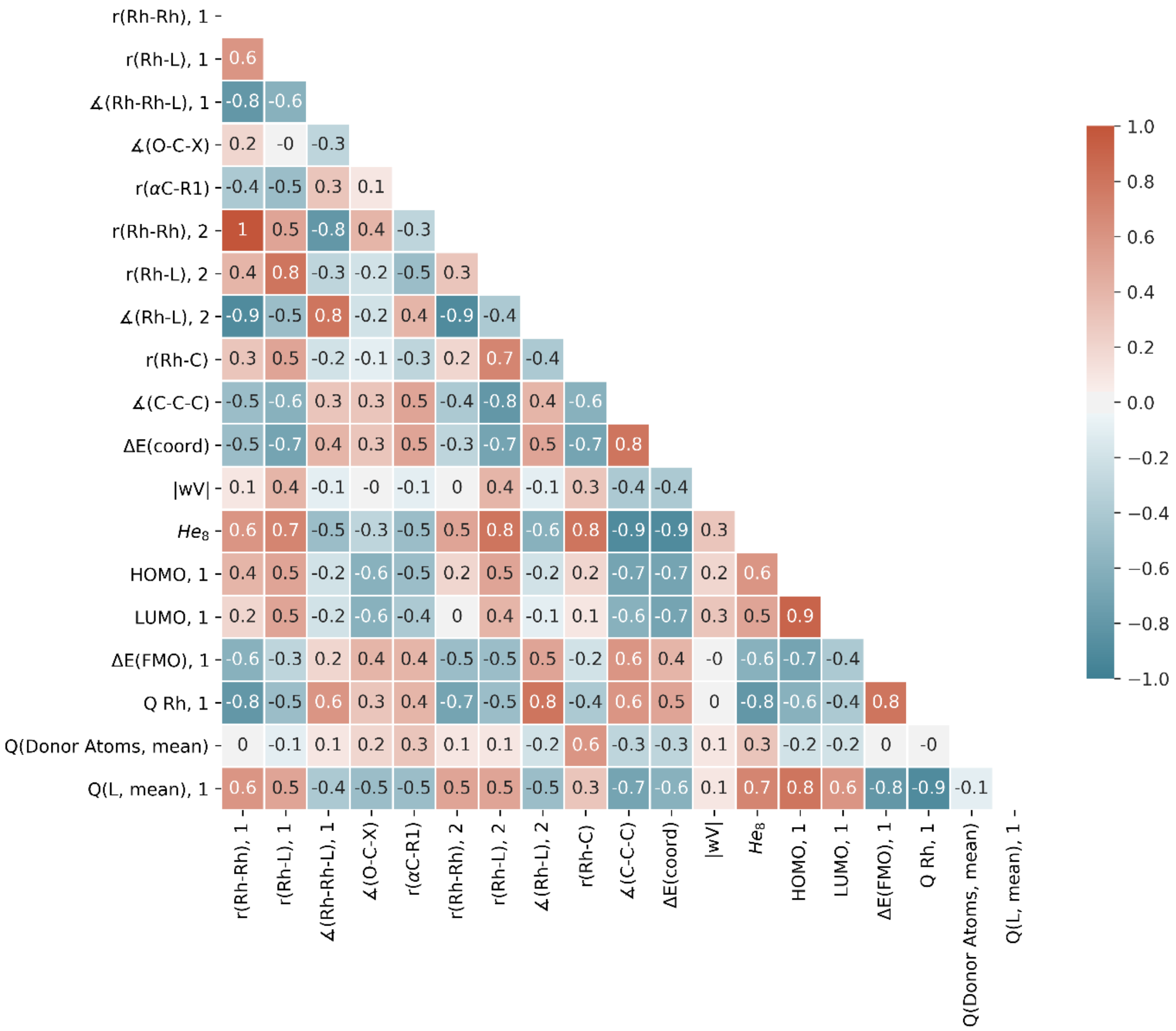

Figure S6. Correlation map for calculated parameters

Principal Component Analysis of Database

PCA (LAPACK implementation, single-value decomposition) was then performed with the sklearn (0.19.2) python (3.7.0) package using the computed chemical descriptors and the python code outlined below.

```
# Required packages for PCA decomposition
import numpy as np
from sklearn.decomposition import PCA

# PCA decomposition
pca = PCA(n_components = 3)
principal_components = pca.fit_transform(x)
```

A PCA decomposition was then fit using sklearn (0.20.3) in python (3.7.3) for all the captured and scaled descriptors. PCA is a method of identifying patterns in datasets with many dimensions (variables).[17,18] PCA performs dimension reduction by converting correlated variables (parameters/descriptors) into sets of orthogonal components that represent most of the variation in the original data. The principal components can then be plotted in two/three dimensions and cluster data based on variance in the original data. This means the integrity of the chemical descriptors is retained, as the underlying data are not manipulated, and catalysts with similar properties will be neighbours, while dissimilar catalysts will be distant in the resulting PCA plot. The benefit of PCA is that the result is representative of the original chemical observations. This is because PCA represents the variance between data points, not the data points themselves, so the analysis does not project absolute values.

After capturing the descriptors outlined a relevant subset must be selected for use in principal component analysis, to ensure the subsequent model can be reliably interpreted and deconvoluted. This means that the model must be generated from descriptors that can be related to chemical features important to the properties of the catalyst. Inclusion of redundant descriptors will make the PCA model harder to interpret as they will not contribute clearly to clustering and have low descriptor loadings which indicates low covariance with a principal component. Low descriptor loadings will reduce the quality of the PCA plot by lowering the amount of variance captured across the first three principal components. The most easily interpreted model will be the model that uses the fewest descriptors to clearly cluster catalysts with similar properties, meaning the ultimate decision on the correct number of parameters is qualitative.

To simplify the number of PCA plots that need to be analysed manually the best solution for each discrete number of descriptors can be calculated (Figure S7). PCA results can be superficially ranked by the total variance captured and by the mean squared error of projection (the amount of information lost through PCA), and the top solution for *n* descriptors is selected by the highest percentage variance captured (for solutions, see Table S5). For qualitative and unsupervised analyses there is often no correct number of parameters for a model, instead models must also be ranked by their interpretability. Models that give even parameter loadings are favoured over those that bias results towards a fewer number of parameters, as they are often better at highlighting complex relationships in the original data.[17]

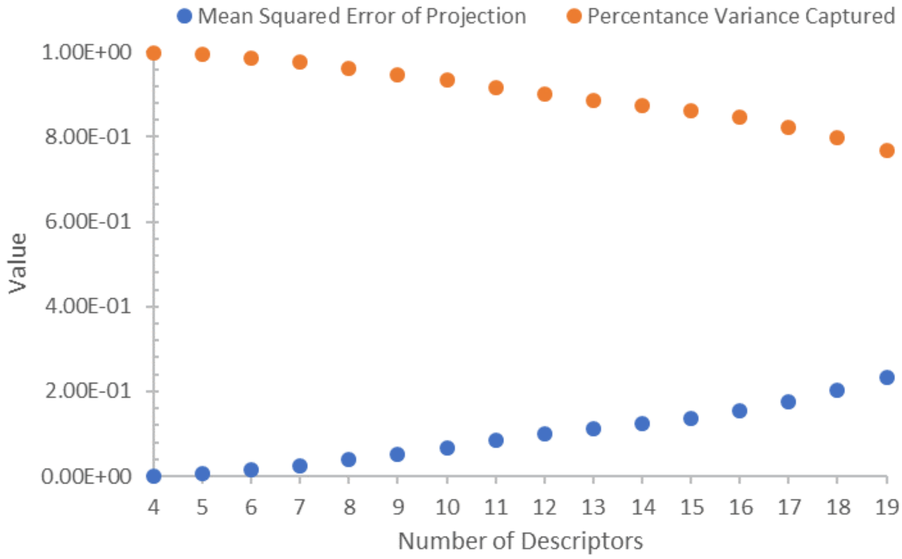

Figure S7. Metrics for optimal PCA solutions of *n* descriptors.

The percentage of variance captured decreases, and the mean squared error of projection increases, as more descriptors are added to the PCA model. This is because the solutions with fewer descriptors have less information to capture and can effectively be represented with only three principal components. While a PCA model that captures 100% of the available information looks appealing from a metric point of view, it does not consider the quality of the clustering in the model or how relevant the information captured is. The next step is to evaluate the PCA plots for their interpretability in the context of dirhodium(II) chemistry (Figure S8 and S9).

Analysis of the first two principal components (PC1 and PC2) (Figure S8) shows that for all combinations of *n* descriptors catalysts are separated by their ligand donor atoms (either *O,O* or *N,O*) by PC1. Further separation of catalyst sub-types begins along PC1 once 11 descriptors are being utilised, highlighting complexes **4y**, **4z** and **4aa** (acyclic *N,O* ligands). PC2 clusters the most electron-withdrawing carboxylate ligands (*O,O*) **3e**, **3f**, **3h** and **3i** immediately, but it does not separate complexes **3g**, **3o** or **3p** from the bulk carboxylate cluster again until 11 descriptors are used.

Analysis of the first and third principal components (PC1 and PC3) (Figure S9) shows that separation of complexes by ligand donor atoms does not fully occur until 6 descriptors are used, allowing the formation of discrete clusters when comparing PC1 and PC3. Complexes **4y**, **4z** and **4aa** are fully separated from the bulk carboxamidate (*N,O*) cluster with 11 descriptors, and when 13 descriptors are added the carboxamidate complexes featuring sterically demanding substituents (**4d**, **4g**, **4m** and **4s**) are highlighted in a new cluster by PC3.

For both series of plots, for PC1/PC2 and PC1/PC3, the clusters begin to disperse, and clustering boundaries become less clear when all the available descriptors are added to the PCA model, which is consistent with lower amounts of captured variance and increasing errors from projection. While the PCA models with greater numbers of descriptors are not bad, models using between 11 and 15 descriptors have the most well-defined clusters and are therefore easier to interpret.

Table S5. Descriptors for each optimal solution

n	Descriptors	Mean Squared Error of Projection	% Variance Captured
4	r(Rh-Rh) **2**, HOMO, **1**, LUMO, **1**, ΔE(FMO)	1.82E-31	99.9
5	r(Rh-Rh) **1**, r(Rh-Rh) **2**, HOMO, **1**, LUMO, **1**, ΔE(FMO)	0.0049	99.5
6	r(Rh-Rh) **1**, r(Rh-Rh) **2**, HOMO, **1**, LUMO, **1**, Q Rh, **1**, Q(L, mean), **1**	0.014	98.6
7	r(Rh-Rh) **1**, ∡(O-C-X), r(Rh-Rh) **2**, HOMO, **1**, LUMO, **1**, Q Rh, Q(L, mean), **1**	0.0234	97.7
8	r(Rh-Rh) **1**, ∡(O-C-X), r(Rh-Rh) **2**, ∡(Rh-Rh-L) Carbene, HOMO, **1**, LUMO, **1**, Q Rh, **1**, Q(L, mean), **1**	0.038	96.2
9	r(Rh-Rh) **1**, ∡(Rh-Rh-L) **1**, r(Rh-Rh) **2**, ∡(Rh-Rh-L) **2**, HOMO, **1**, LUMO, **1**, ΔE(FMO), Q Rh, **1**, Q(L, mean), **1**	0.0516	94.8
10	r(Rh-Rh) **1**, ∡(Rh-Rh-L) **1**, ∡(O-C-X), r(Rh-Rh) **2**, ∡(Rh-Rh-L) **2**, HOMO, **1**, LUMO, **1**, ΔE(FMO), Q Rh, **1**, Q(L, mean), **1**	0.0662	93.4
11	r(Rh-Rh) **1**, r(Rh-Rh) **2**, ∡(Rh-Rh-L) **2**, r(Rh-C), ∡(C-C-C), ΔE(coord), He_8 Ring Interaction, HOMO, **1**, LUMO, **1**, Q Rh, **1**, Q(L, mean), **1**	0.0846	91.5
12	r(Rh-Rh) **1**, ∡(O-C-X), r(Rh-Rh) **2**, ∡(Rh-Rh-L) **2**, ∡(C-C-C), ΔE(coord), He_8 Ring Interaction, HOMO, **1**, LUMO, **1**, ΔE(FMO), Q Rh, **1**, Q(L, mean), **1**	0.0993	90.1
13	r(Rh-Rh) **1**, ∡(Rh-Rh-L) **1**, ∡(O-C-X), r(Rh-Rh) **2**, ∡(Rh-Rh-L) **2**, ∡(C-C-C), ΔE(coord), He_8 Ring Interaction, HOMO, **1**, LUMO, **1**, ΔE(FMO), Q Rh, **1**, Q(L, mean), **1**	0.1132	88.7
14	r(Rh-Rh) **1**, ∡(Rh-Rh-L) **1**, Rh-L Angle, ∡(O-C-X), r(Rh-Rh) **2**, r(Rh-L) **2**, ∡(Rh-Rh-L) **2**, ∡(C-C-C), ΔE(coord), He_8 Ring Interaction, HOMO,**1** , ΔE(FMO), Q Rh, **1**, Q(L, mean), **1**	0.1253	87.4
15	r(Rh-Rh) **1**, Rh-L BL, ∡(Rh-Rh-L) **1**, ∡(O-C-X), r(Rh-Rh) **2**, r(Rh-L) **2**, ∡(Rh-Rh-L) **2**, ∡(C-C-C), ΔE(coord), He_8 Ring Interaction, HOMO, **1**, LUMO, **1**, ΔE(FMO), Q Rh, **1**, Q(L, mean), **1**	0.1373	86.3
16	r(Rh-Rh) **1**, Rh-L BL, ∡(Rh-Rh-L) **1**, ∡(O-C-X), r(Rh-Rh) **2**, r(Rh-L) **2**, ∡(Rh-Rh-L) **2**, r(Rh-C), ∡(C-C-C), ΔE(coord), He_8 Ring Interaction, HOMO, **1**, LUMO, **1**, ΔE(FMO), Q Rh, **1**, Q(L, mean), **1**	0.1537	84.6
17	r(Rh-Rh) **1**, ∡(Rh-Rh-L) **1**, Rh-L Angle, ∡(O-C-X), r(Rh-Rh) **2**, r(Rh-L) **2**, ∡(Rh-Rh-L) **2**, r(Rh-C), ∡(C-C-C), ΔE(coord), He_8 Ring Interaction, HOMO, **1**, LUMO, **1**, ΔE(FMO), Q Rh, **1**, Q Ligand Donor Atoms, Q(L, mean), **1**	0.1764	82.4
18	r(Rh-Rh) **1**, r(Rh-L) **1**, ∡(Rh-Rh-L) **1**, ∡(O-C-X), r(αC-R^1) , r(Rh-Rh) **2**, r(Rh-L) **2**, ∡(Rh-Rh-L) **2**, r(Rh-C), ∡(C-C-C), ΔE(coord), He_8 Ring Interaction, HOMO, **1**, LUMO, **1**, ΔE(FMO), Q Rh, **1**, Q Ligand Donor Atoms, Q(L, mean), **1**	0.203	79.7
19	All available descriptors	0.2328	76.7

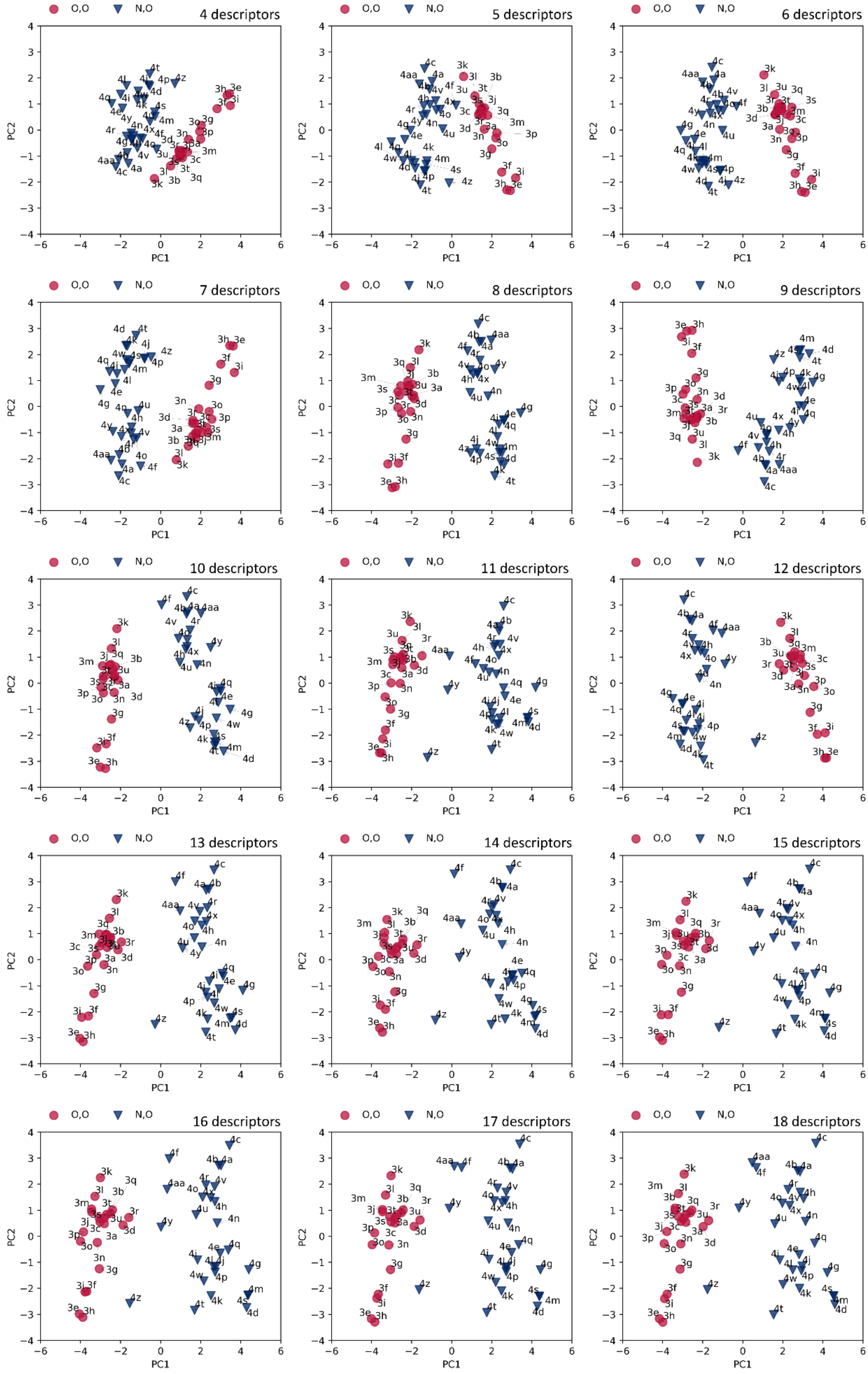

Figure S8. PC1/PC2 plots for the optimal solutions for *n* descriptors.

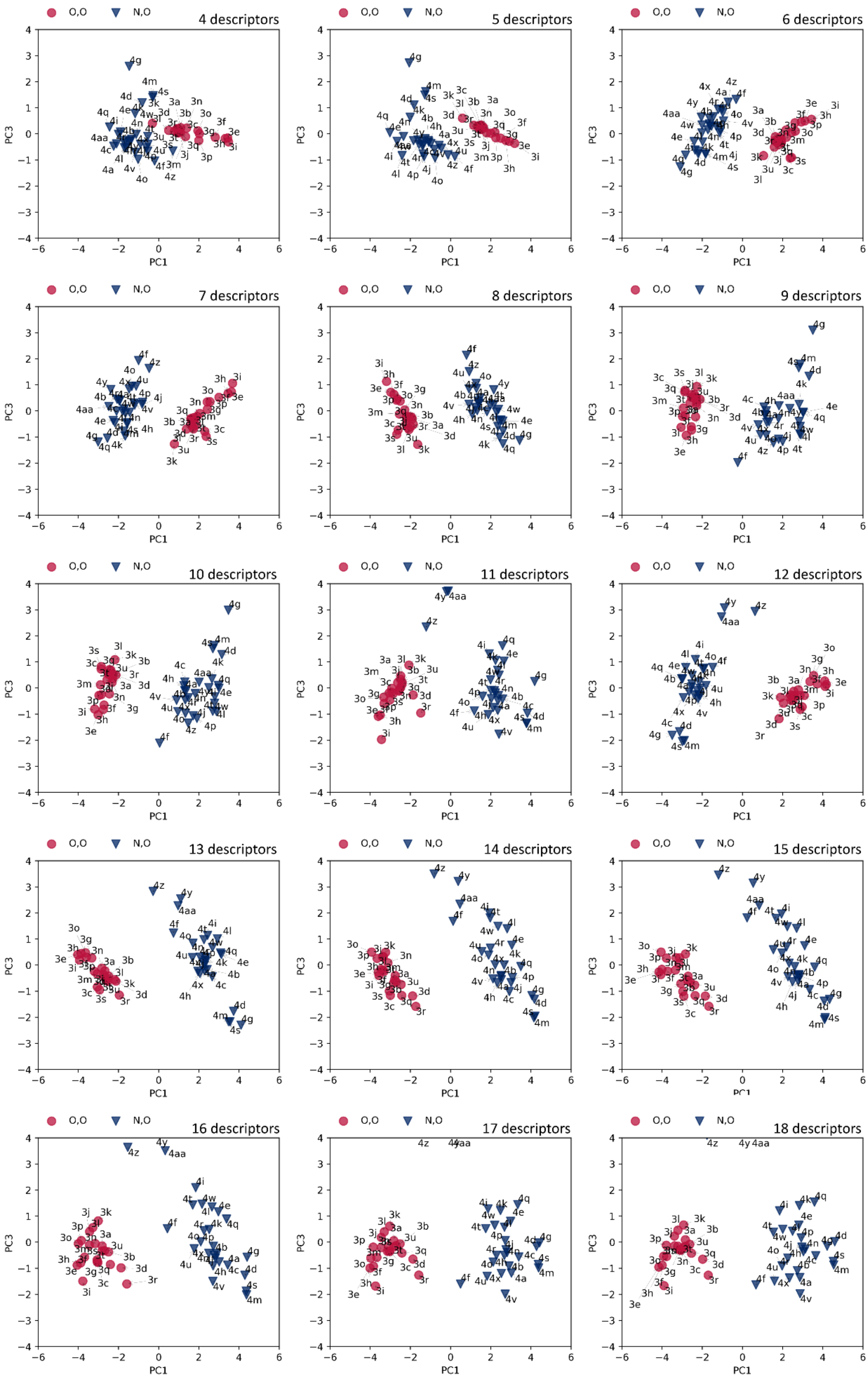

Figure S9. PC1/PC3 plots for the optimal solutions for *n* descriptors.

For the solutions using 11 to 15 descriptors, most of the main catalyst groups are captured by discrete clusters (*O,O* vs. *N,O*, EDG-substituents, EWG-substituents, acyclic *N,O* ligands and sterically demanding *N,O* ligands), but the sterically demanding carboxylate complexes are not represented by a distinct cluster. An extra parameter, |wV|, was added to the *n* = 13 descriptor PCA model to create a 14 descriptor solution (Figure S10 and Figure 4) that forms a new cluster for these bulky *O,O* ligands (**3d**, **3r**, **3s**, **3t** and **3u**). Comparative models with 11, 12, 13, 14 and 15 descriptors were also generated, but the 14 descriptor model gave the best clustering performance. While |wV| was not chosen in the original 14 descriptor PCA model, it was included in several of the top *n* = 14 enumerated solutions, further highlighting the importance of manually evaluating the generated models. |wV| is also a chemically useful descriptor as it contains no electronic information and represents steric contributions only, meaning the descriptor could also be desirable on that basis alone.

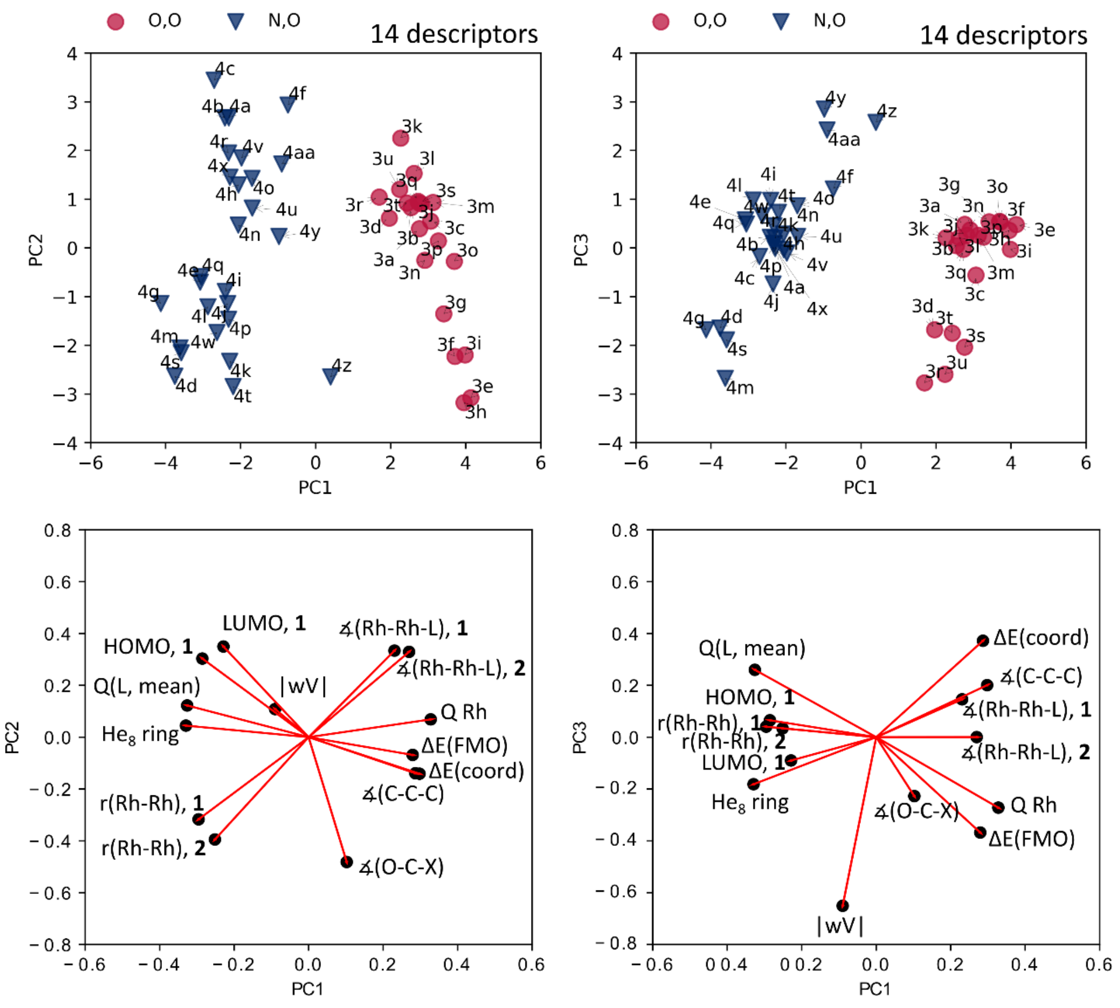

Figure S10. Optimal solution for the PCA of dirhodium(II) catalysts capturing 85.5% total variance. PC1/PC2/PC3 explained variance: 53.6, 21.7 and 10.5%. Mean squared error loss from projection: 0.142.

Following analysis of the PCA scores and loadings plots (Figure S10, Table S6 and S7), it became clear that many of the descriptors capture information on only a few features of each catalyst subset, creating an underlying trend that drives the distinct clustering observed. For example, all the large, aliphatic carboxylate ligands are separated from the largest cluster of carboxylate ligands by greater |wV| and He_8 values on PC3 (Figures S10). Complexes bearing carboxylate ligands are separated from carboxmidate ligands by r(Rh-Rh), and 5- and 6/7- membered cyclic *N,O* ligands are separated ∡(Rh-Rh-L) and He_8 (Figure S10). These contributions illustrate the usefulness of PCA to highlight complex chemical relationships, from appropriate chemical descriptors, where many complexes have remarkably similar values for many other parameters.

Table S6. Descriptor loadings for PC1/2/3 capturing 53.6, 21.7 and 10.5% variance respectively.

Descriptor	Loadings PC1	Loadings PC2	Loadings PC3
% contribution to variance	53.6	21.7	10.5
r(Rh-Rh), **1**	-0.2955	-0.3171	0.0404
∡(Rh-L), **1**	0.2306	0.3339	0.1460
∡(O-C-X), **1**	0.1023	-0.4817	-0.2274
r(Rh-Rh) **2**	-0.2516	-0.3947	0.0341
∡(Rh-Rh-L) **2**	0.2697	0.3292	0.0003
∡(C-C-C)	0.2978	-0.1411	0.2007
ΔE(coord)	0.2866	-0.1394	0.3720
He_8 Ring Interaction	-0.3294	0.0445	-0.1815
HOMO, **1**	-0.2854	0.3033	0.2599
LUMO, **1**	-0.2287	0.3498	0.0652
ΔE(FMO)	0.2793	-0.0685	-0.0900
Q Rh, **1**	0.3278	0.0686	-0.3680
Q(L, mean), **1**	-0.3260	0.1223	-0.2720
|wV|	-0.0901	0.1084	-0.6506

Table S7. PCA Eigenvalues for PC1/2/3

Complex	PC1	PC2	PC3
3a	2.765124	0.394087	0.484166
3b	2.540759	0.826934	0.045106
3c	3.063959	0.547643	-0.55779
3d	1.964327	0.611458	-1.67993
3e	4.135164	-3.07732	0.48039
3f	3.709259	-2.22617	0.528501
3g	3.415276	-1.35453	0.537182
3h	3.952224	-3.17644	0.358793
3i	3.981831	-2.19594	-0.03057
3j	2.816006	0.904917	0.259393
3k	2.262869	2.254427	0.206891
3l	2.618915	1.529706	0.209988
3m	3.126386	0.931589	0.256251
3n	2.910799	-0.25628	0.367238
3o	3.692856	-0.27468	0.545152
3p	3.271487	0.143183	0.224148
3q	2.732144	0.955249	-0.02999
3r	1.690642	1.041756	-2.77287
3s	2.757774	0.918259	-2.03934
3t	2.429197	0.918512	-1.74944
3u	2.235113	1.202976	-2.59396
4a	-2.31806	2.673761	0.080818
4aa	-0.90338	1.722572	2.42053
4b	-2.42068	2.666118	0.217413
4c	-2.70736	3.442609	-0.17431
4d	-3.75862	-2.63471	-1.64474
4e	-3.08175	-0.70591	0.577585
4f	-0.73693	2.934292	1.212598
4g	-4.12887	-1.14019	-1.6823
4h	-2.05698	1.294935	-0.03218
4i	-2.40733	-0.89312	0.97583
4j	-2.34399	-1.14344	-0.74184
4k	-2.29413	-2.33117	0.200509
4l	-2.87215	-1.21016	0.982162
4m	-3.61938	-2.05115	-2.67747
4n	-2.06685	0.469549	0.283543
4o	-1.69553	1.427121	0.866271
4p	-2.32495	-1.46413	0.096215
4q	-3.05464	-0.58895	0.491496
4r	-2.31969	1.94737	0.45642
4s	-3.58574	-2.15338	-1.88819
4t	-2.20451	-2.84644	0.730663
4u	-1.69042	0.81985	0.236748
4v	-1.97567	1.848575	-0.10632
4w	-2.63137	-1.73973	0.658611
4x	-2.29055	1.450535	-0.01318
4y	-0.97945	0.235402	2.846985
4z	0.396875	-2.64954	2.576822

A separate PCA decomposition was also conducted on the *O,O* and *N,O* sub-classes of Rhodium(II) complexes to investigate the variation in catalyst properties for each ligand donor atom type. PCA of the *O,O* sub-class (Figure S11) separates complexes by electronic properties along PC1 for this new model, with complexes bound to electron-withdrawing ligands (**3e** – **3i**) between PC1: 4 – 6, and by steric properties along PC2 with the complexes bound to sterically demanding ligands (**3d**, **3r** – **3u**) between PC2: 1 – 4. These trends are similar to the full PCA model but with greater distance between each complex highlighting the vast range variance of ligand properties within the carboxylate cluster.

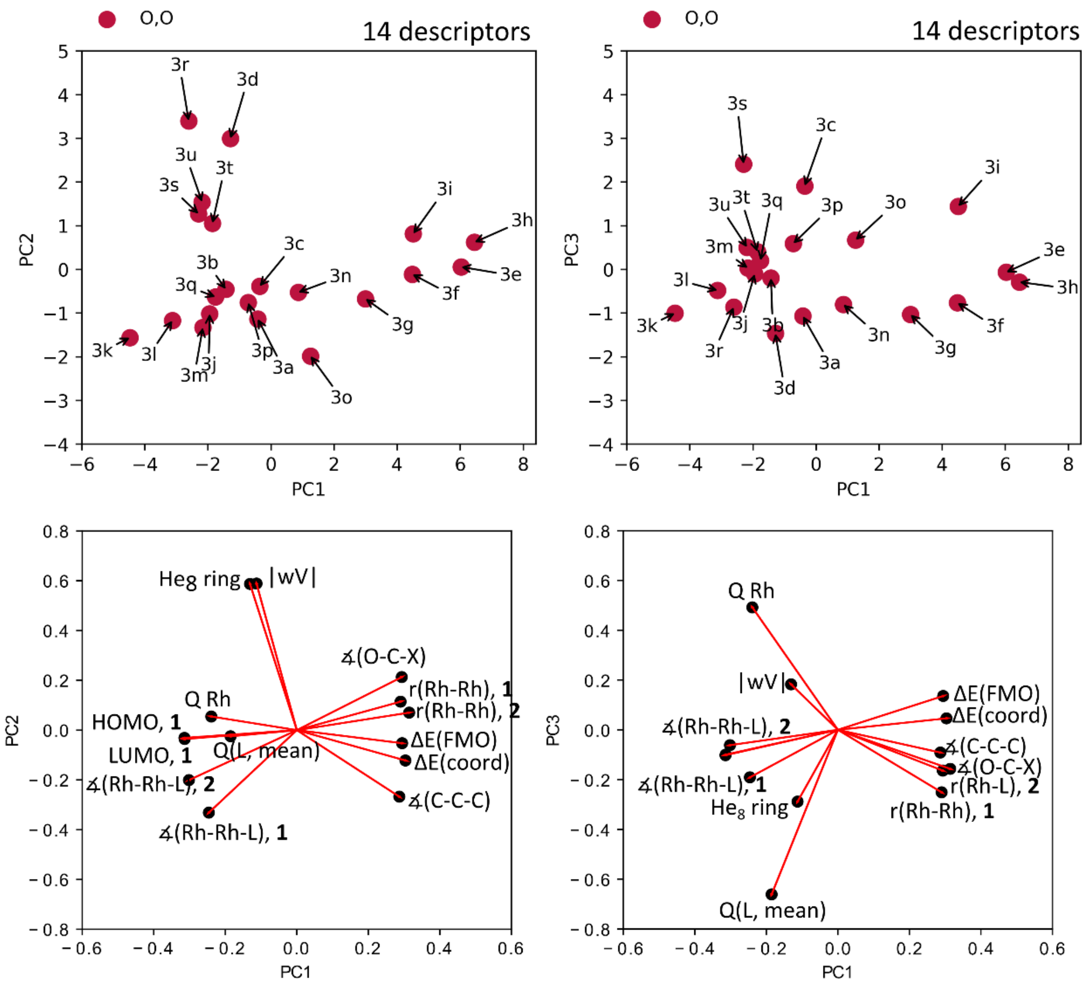

Figure S11. Optimal solution for the PCA of O,O dirhodium(II) catalysts capturing 87.4% total variance. PC1/PC2/PC3 explained variance: 66.6, 13.8 and 7.0%. Mean squared error loss from projection: 0.126.

PCA of the *N,O* sub-class (Figure S12) separates complexes based on their cyclic backbone ring size (5 or 6/7- membered rings) and their electronic properties along PC1. For example, **4t**, a 5-membered

lactam ring substituted with fluorine atoms, is at the peak of the right-hand cluster (PC1: 3) and the urea **4q**, a 5-membered lactam ring, is on the opposing edge (PC1: 0). This trend is mirrored in the left-hand cluster with **4u**, a 6-membered lactam ring substituted with fluorine atoms, is located at PC1: -1 and the catalyst on the opposing edge is the unsubstituted 6-membered lactam **4c**. PC2 separates the *N*,*O* complexes by steric factors, with the acyclic ligands located at the top of the plot (PC2: 2 – 6) and the sterically-demanding complexes at the bottom (PC2: -2 to -4). Analysis of the carboxamidate cluster alone gives greater resolution of electronic ligand effects, when compared to the full model, due to the lower density of clustering, and shows that the Lewis acidity of carboxamidate complexes is also captured by the model.

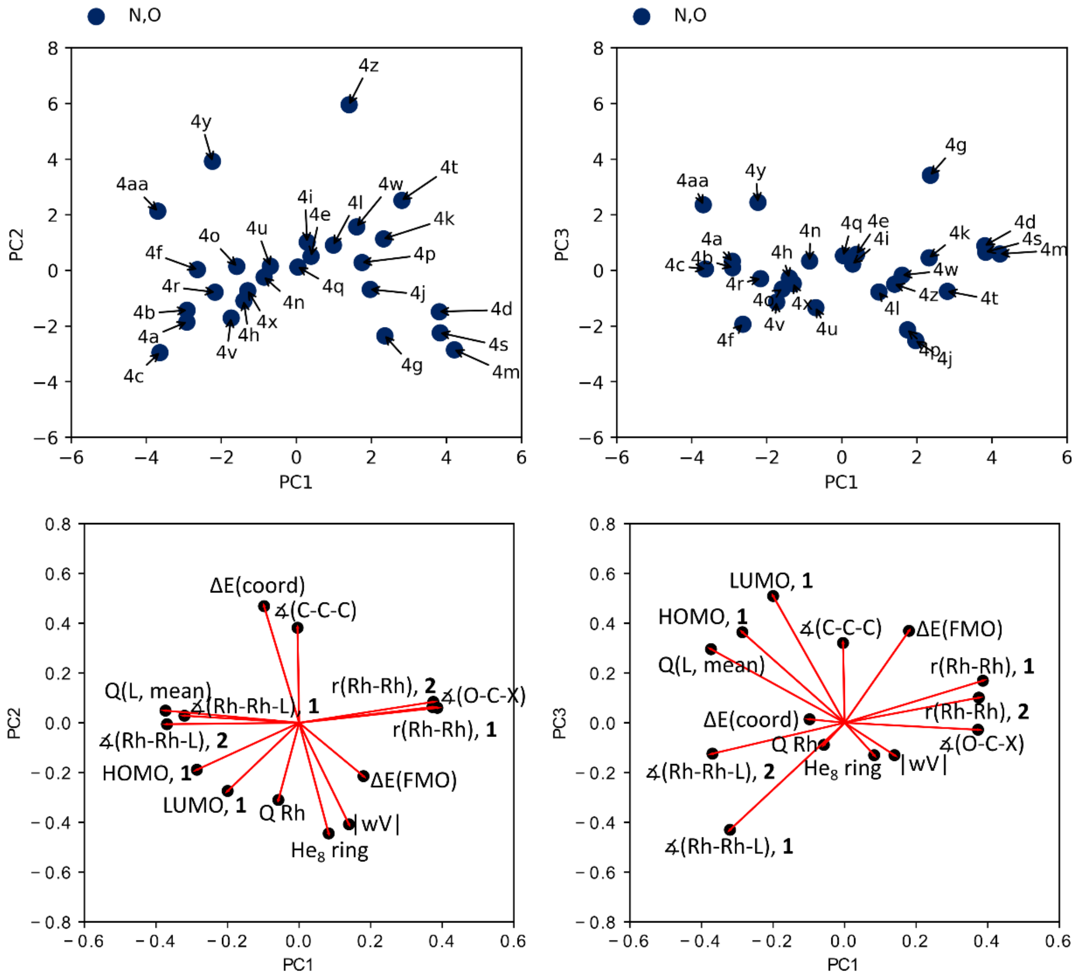

Figure S12. Optimal solution for the PCA of N,O dirhodium(II) catalysts capturing 80.3% total variance. PC1/PC2/PC3 explained variance: 39.5, 28.7 and 12.1%. Mean squared error loss from projection: 0.196.

Relationship with Experimental Data

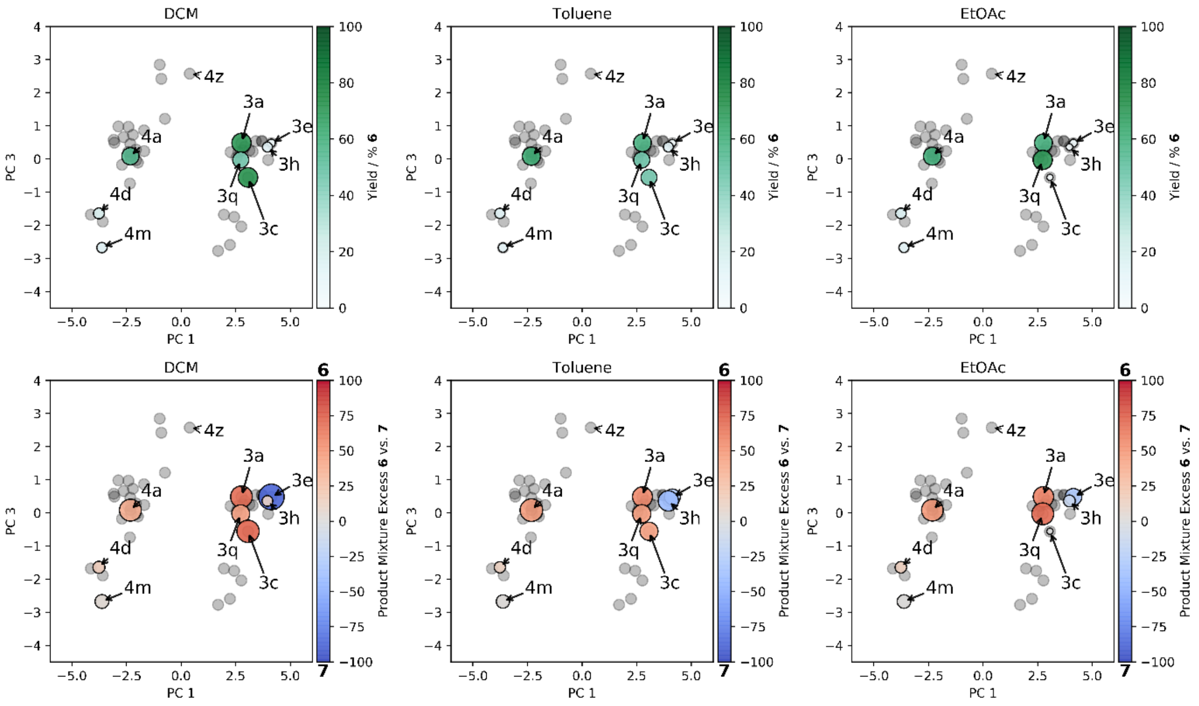

Figure S13. Overlay of reaction outcomes onto PC1/PC3 plot. Top: HPLC yield of the β-lactam **6** as a function of catalyst and solvent shown within the context of the catalyst map (green, circles scaled according to yield, with darker shades also corresponding to higher % yield of 6); catalysts that were not investigated experimentally are also shown (grey, **4z** labelled). Bottom: Ratio of HPLC peak areas corresponding to the alternative products **6** and **7** (circles scaled according to conversion, with red favouring **6** and blue favouring **7**).

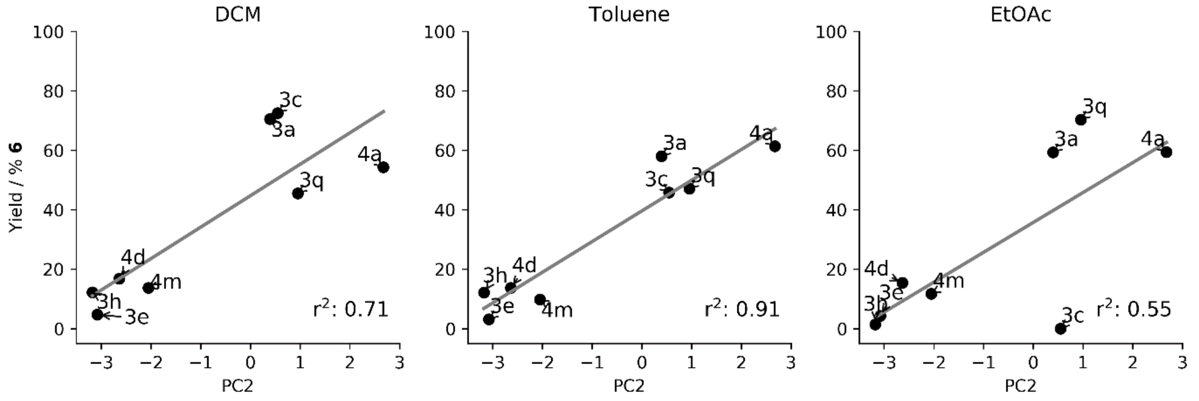

Figure S14. Correlation of PC2 with the yield of **6** in all three reaction solvents.

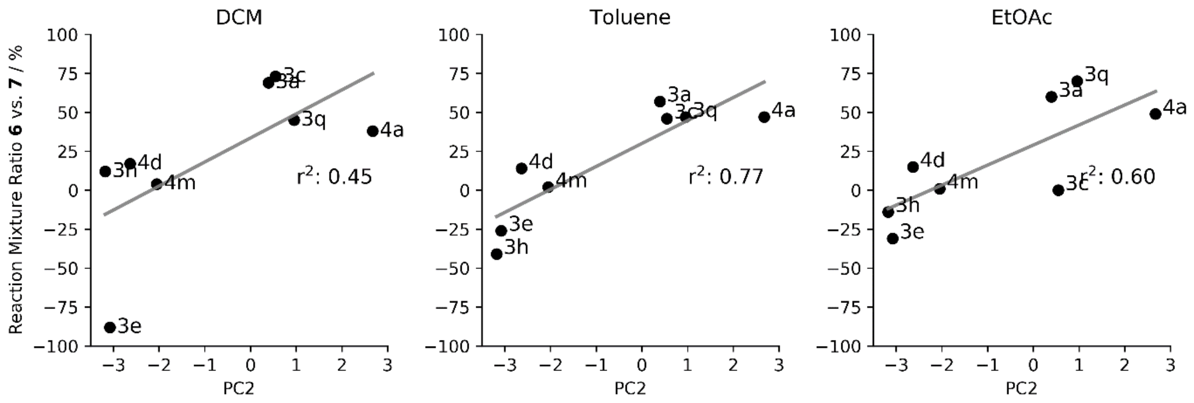

Figure S15. Correlation of PC2 with selectivity between the formation of **6** or **7** (+100% represents exclusive formation of **6** and -100% represents exclusive formation of **7**).

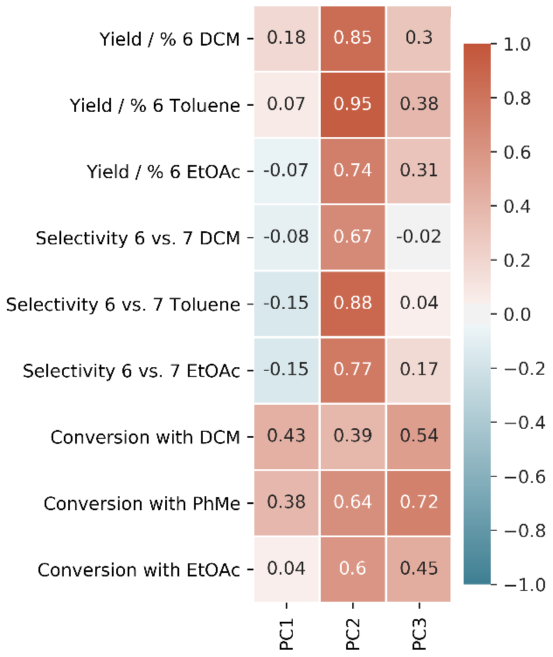

Figure S16. Pearson R correlations between the experimental data and PC1, PC2 or PC3.

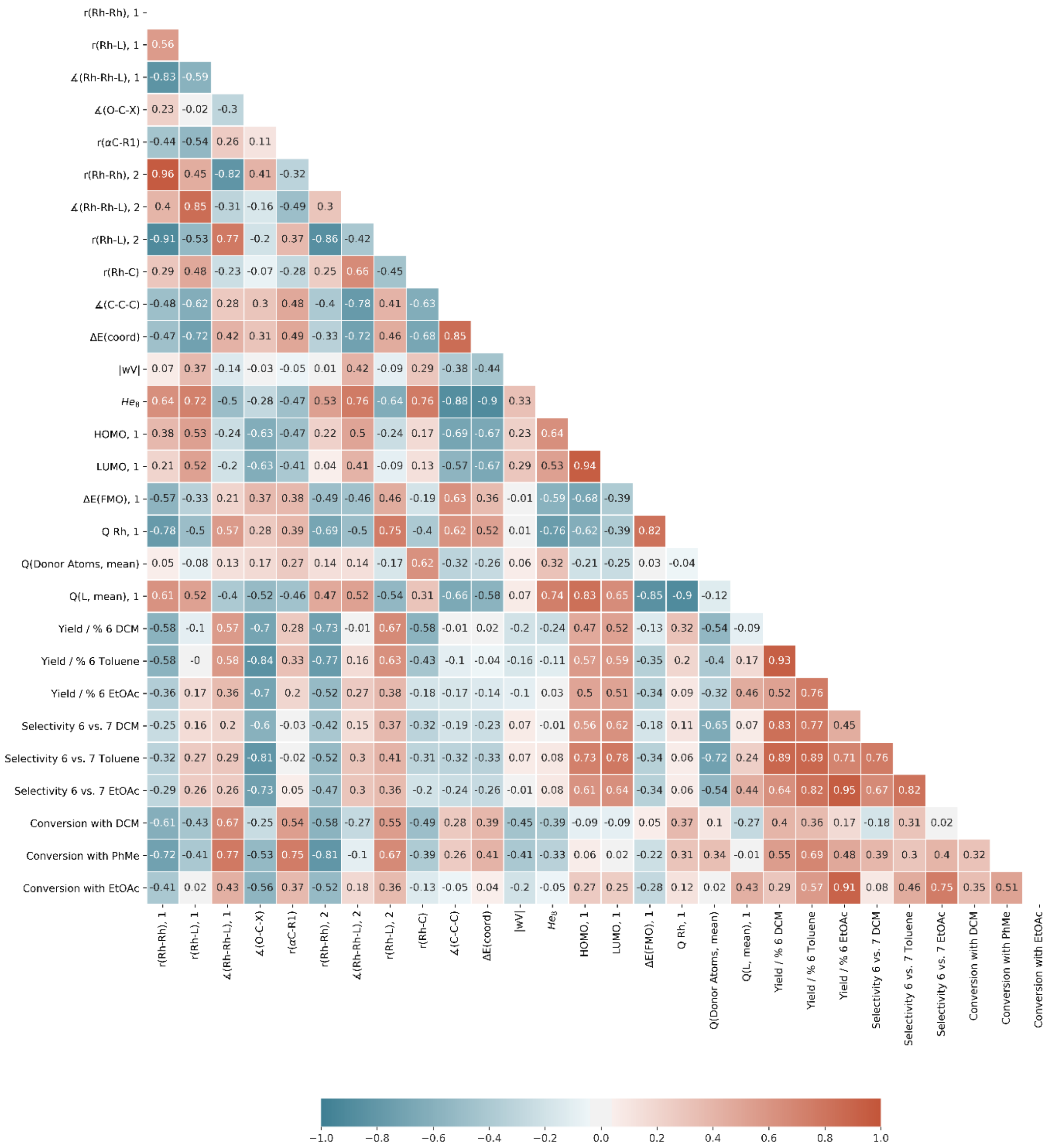

Figure S17. Pearson R correlations between the experimental data and all calculated descriptors.

References

1 G. Nakatsuji, H., Caricato, M., Li, X., Hratchian, H. P., Izmaylov, A. F., Bloino, J., Zheng, M. Sonnenberg, J. L., Hada, M., Ehara, M., Toyota, K., Fukuda, R., Hasegawa, J., Ishida, J. E. Nakajima, T., Honda, Y., Kitao, O., Nakai, H., Vreven, T., Montgomery, J. A., Jr., Peralta, R. Ogliaro, F., Bearpark, M., Heyd, J. J., Brothers, E., Kudin, K. N., Staroverov, V. N., Kobayashi, M. Normand, J., Raghavachari, K., Rendell, A., Burant, J. C., Iyengar, S. S., Tomasi, J., Cossi, J. Rega, N., Millam, N. J., Klene, M., Knox, J. E., Cross, J. B., Bakken, V., Adamo, C., Jaramillo, J. W. Gomperts, R., Stratmann, R. E., Yazyev, O., Austin, A. J., Cammi, R., Pomelli, C., Ochterski, J. J. Martin, R. L., Morokuma, K., Zakrzewski, V. G., Voth, G. A., Salvador, P., Dannenberg, D. J. Dapprich, S., Daniels, A. D., Farkas, Ö., Foresman, J. B., Ortiz, J. V., Cioslowski, J., Fox and W. CT. Gaussian, Inc., 2009.

2 A. D. Becke, *Physical Review A*, 1988, **38**, 3098–3100.

3 J. P. Perdew, *Physical Review B*, 1986, **33**, 8822–8824.

4 J. P. Perdew, *Physical Review B*, 1986, **34**, 7406.

5 R. Ditchfield, W. J. Hehre and J. A. Pople, *J. Chem. Phys*, 1971, **54**, 724.

6 W. J. Hehre, R. Ditchfield and J. A. Pople, *J. Chem. Phys*, 1972, **56**, 2257.

7 P. C. Hariharan and J. A. Pople, *The Influence of Polarization Functions on Molecular Orbital Hydrogenation Energies*, Springer-Verlag, 1973, vol. 28.

8 M. M. Francl, W. J. Pietro, W. J. Hehre, J. S. Binkley, M. S. Gordon, D. J. Defrees and J. A. Pople, *The Journal of Chemical Physics*, 1982, **77**, 3654.

9 D. Andrae, U. Häußermann, M. Dolg, H. Stoll and H. Preub, *Theoretica Chimica Acta Energy-adjusted ab initio pseudopotentials for the second and third row transition elements*, 1990, vol. 77.

10 K. Gilbert, *PCModel*, Bloomington, IN, 2004.

11 K. Liao, W. Liu, Z. L. Niemeyer, Z. Ren, J. Bacsa, D. G. Musaev, M. S. Sigman and H. M. L. Davies, *ACS Catalysis*, 2018, **8**, 678–682.

12 N. Fey, A. C. Tsipis, S. E. Harris, J. N. Harvey, A. G. Orpen and R. A. Mansson, *Chemistry - A European Journal*, 2006, **12**, 291–302.

13 S. Aguado-Ullate, S. Saureu, L. Guasch and J. J. Carbó, *Chemistry - A European Journal*, 2012, **18**, 995–1005.

14 T. Piou, F. Romanov-Michailidis, M. Romanova-Michaelides, K. E. Jackson, N. Semakul, T. D. Taggart, B. S. Newell, C. D. Rithner, R. S. Paton and T. Rovis, *Journal of the American Chemical Society*, 2017, **139**, 1296–1310.

15 A. V. Brethomé, S. P. Fletcher and R. S. Paton, *ACS Catalysis*, 2019, **9**, 2313–2323.

16 N. Fey, A. Koumi, A. V Malkov, J. D. Moseley, B. N. Nguyen, N. G. Tyler and C. E. Willans, *Dalton Transactions*, 2020, DOI: 10.1039/D0DT01694B.

17 T. Hastie, R. Tibshirani and J. Friedman, *The Elements of Statistical Learning - Second Edition*, Springer-Verlag, 2013.

18 J. VanderPals, *Python Data Science Handbook*, O'Reilly, California, First Ed., 2016.
